# Enabling growth-decoupled *Komagataella phaffii* recombinant protein production based on the methanol-free P_DH_ promoter

**DOI:** 10.3389/fbioe.2023.1130583

**Published:** 2023-03-23

**Authors:** Núria Bernat-Camps, Katharina Ebner, Veronika Schusterbauer, Jasmin Elgin Fischer, Miguel Angel Nieto-Taype, Francisco Valero, Anton Glieder, Xavier Garcia-Ortega

**Affiliations:** ^1^ Department of Chemical, Biological, and Environmental Engineering, School of Engineering, Universitat Autònoma de Barcelona, Bellaterra, Spain; ^2^ Austrian Centre of Industrial Biotechnology (ACIB), Graz, Austria; ^3^ Bisy GmbH, Hofstaetten, Austria

**Keywords:** *Komagataella phaffii*, PDH, recombinant protein production, methanol-free expression system, growth-decoupled bioprocess, promoter regulation, *Pichia pastoris*

## Abstract

The current transition towards the circular bioeconomy requires a rational development of biorefineries to sustainably fulfill the present demands. The use of *Komagataella phaffii* (*Pichia pastoris*) can meet this challenge, since it has the capability to use crude glycerol as a carbon-source, a by-product from the biodiesel industry, while producing high- and low-added value products. Recombinant protein production (RPP) using *K. phaffii* has often been driven either by the methanol induced *AOX1* promoter (P_*AOX1*_) and/or the constitutive *GAP* promoter (P_*GAP*_). In the last years, strong efforts have been focused on developing novel expression systems that expand the toolbox variety of *K. phaffii* to efficiently produce diverse proteins that requires different strategies. In this work, a study was conducted towards the development of methanol-free expression system based on a heat-shock gene promoter (P_DH_) using glycerol as sole carbon source. Using this promoter, the recombinant expression is strongly induced in carbon-starving conditions. The classical P_*GAP*_ was used as a benchmark, taking for both strains the lipase B from *Candida antarctica* (CalB) as model protein. Titer of CalB expressed under P_DH_ outperformed P_*GAP*_ controlled expression in shake-flask cultivations when using a slow-release continuous feeding technology, confirming that P_DH_ is induced under pseudo-starving conditions. This increase was also confirmed in fed-batch cultivations. Several optimization rounds were carried out for P_DH_ under different feeding and osmolarity conditions. In all of them the P_DH_ controlled process outperformed the P_*GAP*_ one in regard to CalB titer. The best P_DH_ approach reached 3.6-fold more specific productivity than P_*GAP*_ fed-batch at low μ. Compared to the optimum approach for P_*GAP*_-based process, the best P_DH_ fed-batch strategy resulted in 2.3-fold higher titer, while the specific productivity was very similar. To summarize, P_DH_ is an inducible promoter that exhibited a non-coupled growth regulation showing high performance, which provides a methanol-free additional solution to the usual growth-coupled systems for RPP. Thus, this novel system emerges as a potential alternative for *K. phaffii* RPP bioprocess and for revaluing crude glycerol, promoting the transition towards a circular economy.

## 1 Introduction

Currently, the circular bioeconomy implementation is usually considered one of the most promising solutions to address the twin challenges of climate change and growing population (Ubando et al., 2020). Ideally, the biorefinery concept enables sustainable economic growth by facilitating the production of a broad spectrum of products from different renewable feedstocks using physico-chemical and biological procedures (Areniello et al., 2022). To develop this circular network, the ability of microorganisms to convert raw substrates into small molecules and proteins is exploited *via* fermentation processes, using either wild-type or engineered organisms (Cherubini, 2010). However, many interesting feedstocks, such as lignocellulosic biomass, usually require enzymatic pre-treatment to become bioaccessible for the microorganism (Tamburino et al., 2022).

In the last decades, *Komagataella phaffii*, a yeast formerly known as *Pichia pastoris,* has become widely used as a cell factory for recombinant protein production (RPP) and metabolites (Juturu and Wu, 2018). Interestingly, *K. phaffii* is an ideal platform to integrate in a biorefinery, at both the feedstock pre-treatment stage and production fermentation stage. In addition to the exceptional ability to metabolize methanol, *K. phaffii* is used to produce high-value added proteins using different cheap substrates, including glycerol derived from biodiesel industry as the sole carbon source (Potvin et al., 2012). Alternatively, *K. phaffii* is also a suitable cell chassis for the synthesis of low-value commodity enzymes, such as cellulases for the upstream feedstock transformation of lignocellulosic biomass (Bernardi et al., 2019; Yang et al., 2019). To facilitate this ubiquitous presence of *K. phaffii* in biorefinery, a wide range of molecular tools are needed to meet each target protein optimal expression strategy. Among those tools investigated over the past years, promoter selection, which has a major impact in both the process development and final product obtained, has generated relevant outcomes (Vogl and Glieder, 2013; Yang and Zhang, 2018; Vogl et al., 2020).

Due to its ability to grow on methanol as sole carbon source, *K. phaffii* owns a set of strong and inducible promoters that regulates tightly the methanol utilization (MUT) pathway. Among them, the alcohol oxidase 1 promoter (P_*AOX1*_) has been widely used for driving the expression of numerous recombinant proteins (Vogl and Glieder, 2013). In terms of regulation, P_*AOX1*_ is tightly repressed when *K. phaffii* is grown on glucose, glycerol or ethanol, and slightly de-repressed upon its depletion. Full induction is only achieved after methanol addition, which allows obtaining high product titers and yields (Ahmad et al., 2014). This inducible regulation enables the uncoupling of growth and protein production, by using glycerol as the carbon source in the batch phase and then switching to methanol, which induces the target protein expression (Looser et al., 2015). This strategy avoids cell burden due to protein overexpression during the biomass generation phase, being especially suitable for the production of toxic proteins (Yang and Zhang, 2018). Although P_*AOX1*_-based expression is widespread for producing efficiently recombinant proteins; the use of methanol, especially for biopharmaceuticals, is often avoided at industrial scale due to storage and handling costs (Karbalaei et al., 2020). Additionally, the higher heat production and O_2_ requirements related to methanol metabolization usually cause an increase in the operational costs (Çalik et al., 2015). Hence, in the last years the focus has shifted towards studying new expression systems that avoid the use of methanol, but still achieving high titers and productivities (Juturu and Wu, 2018; Yang and Zhang, 2018).

An interesting approach for identifying novel methanol-free expression systems is to move away from MUT related promoters, for example by choosing promoters from other metabolic pathways. This would be the case of the glyceraldehyde-3-phosphate promoter (P_*GAP*_) and the phosphoglycerate kinase promoter (P_*PGK*_), which are involved in the glycolysis and gluconeogenesis pathway, respectively. Although both are constitutive, P_*PGK*_ presents a rather weak expression profile, while P_*GAP*_ is considered a strong promoter that can reach similar expression levels as P_*AOX1*_ (Vogl and Glieder, 2013). The use of P_*GAP*_-based expression systems also makes fed-batch cultivations easier, since no transition phase is required to shift the carbon source, making it a commonly used alternative to the P_*AOX1*_ bioprocess (Yang and Zhang, 2018). However, strong and constitutive expression is not always desired, especially when protein folding is the limiting step or RPP can generate cell toxicity (Vogl and Glieder, 2013). Another promising promoter identified from a metabolic pathway is the promoter of the alcohol dehydrogenase 2 (P_*ADH2*_), whose gene is responsible for ethanol consumption (Karaoglan et al., 2016b). Upon induction with ethanol, P_*ADH2*_-based strain achieved similar specific productivity than the P_*AOX1*_-based strain when producing a xylanase under standard cultivation conditions (Karaoglan et al., 2016a). Due to its potential to replace the historical *K. paffii* promoters, several engineered variants have been generated by modifying or replacing the regulatory regions of P_*ADH2*_, achieving up to 4-fold increases in the product to biomass yield (Ergün et al., 2019; Erden-Karaoğlan et al., 2022). Although ethanol is less hazardous than methanol, these processes present the same disadvantage of having longer fed-batch cultivations, due to the adaptation phase to the new carbon source and the slower specific growth rates (Potvin et al., 2016; Erden-Karaoğlan et al., 2022).

Another option that avoids the use of methanol and has gained great attention is to develop methanol-free P_*AOX1*_-based systems. This is achieved by triggering the methanol activating pathway or inactivating the catabolite repression pathway (Yang and Zhang, 2018). Based on this pathway engineering approach, the development of several methanol-independent processes has been reported in the literature, where transcriptional repressors or genes involved in P_*AOX1*_ regulation were knocked out and/or transcriptional activators were overexpressed (Shen et al., 2016; Wang et al., 2017; Vogl et al., 2018; Dalvie et al., 2022). Interestingly, the producer system designed by Wang et al. (2017) (MF1), which was repressors-deficient together with an overexpression of an activator gene, reached 58.6% of the insulin precursor amount produced with wild-type (WT) P_*AOX1*_ system, but avoiding the use of methanol. This was the highest activation achieved by a P_*AOX1*_-based system without methanol induction (Vogl et al., 2018).

Alternatively, related methylotrophic yeast species are a rich source for investigating novel promoters (Stadlmayr et al., 2010; Vogl et al., 2020). P_*HpFMD*_, as an example, is an orthologous promoter from *Hansenula polymorpha*, which shows derepressed regulation when growing on either glycerol or glucose, and additionally is further inducible by methanol. According to the literature, it is the strongest promoter so far reported in *K. phaffii* when grown on methanol (Vogl et al., 2020), which served to generate a commercial variant named P_DF_. Recently, the P_DF_-based system surpassed the specific production rate (q_p_) of the methanol-free benchmark P_*GAP*_-based system by the factor of nine, when expressing *Candida antarctica* lipase B (CalB) in chemostat cultivations (Fischer et al., 2019; Garrigós-Martínez et al., 2021).

All the expression systems mentioned above were engineered or identified from known highly expressed genes, either from *K. phaffii* or other related yeasts. Currently, the emerging systems biology tools can also ease the identification of novel expression systems. In particular, transcriptomics is a powerful method for identifying upregulated genes under specific conditions (Vogl and Glieder, 2013). This way, the pursuit of novel methanol-free and differently regulated expression systems can be rationally developed. The first genome-wide promoter study used publicly available data obtained from the heterologous microarray hybridization of *K. phaffii* cDNA to a *Saccharomyces cerevisiae* specific microarray, from fed-batch cultivations on glucose, glycerol or methanol. Together with already reported promoters, P_*THI11*_, which regulates the expression of a protein involved in thiamine precursor synthesis, was isolated. The activity of this novel promoter was found to be uninfluenced by any carbon or nitrogen source, but regulated by the thiamine availability (Stadlmayr et al., 2010). The emergence of whole genome sequencing opened the doors for *K. phaffii* specific transcriptome tools. This included specific *K. phaffii* microarray chips (Graf et al., 2008) and whole genome assemblies of multiple *K. phaffii* strains, which build the basis for RNA-Seq based transcriptome studies (De Schutter et al., 2009; Mattanovich et al., 2009; Küberl et al., 2011; Liang et al., 2012).

Using RNA-Seq the genome annotation of *K. phaffii* was further improved and gene expression in glycerol or methanol chemostat cultivations was analyzed (Liang et al., 2012). From this data, a methanol-free strong and constitutive promoter called P_*GCW14*_ was identified, which natively regulates the expression of a potential glycosyl phosphatidyl inositol (GPI)-anchored protein (Liang et al., 2013). Due to the great performance that P_*GCW14*_ exhibited, a commercial variant called P_*UPP*_ was generated and characterized, presenting up to 9-fold more q_p_ than the P_*GAP*_-based strain producing CalB in glycerol-based chemostat cultivations (Garrigós-Martínez et al., 2021). In the work described by Prielhofer et al. (2013), a rational approach was reported to identify novel promoters inducible under glucose-limiting conditions. In this case, the methodology was based on *K. phaffii* specific microarray hybridization of mRNA samples from a glucose chemostat cultivation. The promoter of a high affinity glucose transporter (P_*GTH1*_) was successfully identified, which is repressed on glycerol and highly induced in glucose-limiting conditions (Prielhofer et al., 2013). Finally, in a genome-wide transcriptional response study to different carbon sources, the promoter involved in reactive oxygen species defense, named P_*CAT1*_, was identified for being strongly induced by methanol and oleic acid, reaching similar expression levels as P_*AOX1*_ *.* Interestingly, this promoter also presents a tight repression and de-repression on glucose obtaining high titers and productivities. It can be also found as a commercial variant named P_DC_ (Vogl et al., 2016; Fischer et al., 2019).

Although the number of available promoters for RPP in *K. phaffii* is becoming wider, there are still few options that meet the current trend of developing methanol-independent bioprocess that are not growth-coupled. In this study, the aim was to identify novel methanol-free and tightly regulated promoters in *K. phaffii*, specifically under strict carbon-limiting conditions and without adding any inducer. Using RNA-Seq technology, several genes were observed to be highly expressed when applying low glycerol feeding, which allow cell maintenance but no biomass growth. From the different candidates identified, the promoter region of heat shock protein 12 gene (*HSP12*) was selected as promising to develop a novel expression system. This family of proteins, which are upregulated under general stress conditions, has been extensively studied in fungi (Tiwari et al., 2015). Specifically in *S. cerevisiae*, *HSP12* is tightly repressed by glucose, being expressed in the stationary phase but not when growing (de Groot et al., 2000; Karreman and Lindsey, 2005). In this work, the promoter region of *HSP12* in *K. phaffii* was identified and named as P_DH_. To study its regulation and potential for RPP, the industrially relevant enzyme CalB was used as a reporter protein. The performance of P_DH_-based expression system in different cultivation set-ups was compared to the commonly used P_*GAP*_ and the strong and commercial P_DF_ and P_*UPP*_, all considered as methanol-free benchmarks.

This work will contribute to enlarge the *Pichia* toolbox by providing more alternatives for the efficient production of different kind of proteins. On balance, this can promote the transition towards the circular bioeconomy, thus significantly decreasing our carbon footprint.

## 2 Materials and methods

### 2.1 Glycerol release measurement from FeedBeads^®^

This work has required to run carbon-limited cultures in shake-flask cultivations, which has been performed using the glycerol-releasing FeedBeads^®^ supplied by Kuhner (SMFB12001, Kuhner Shaker, Basel, Switzerland). This slow-release substrate technology is based on silicone matrix discs with the substrate (glycerol or glucose) embedded. They ensure that this technology reduces the negative influences of O_2_ limitation, by-products production and pH-shifts, while also improving the product yields.

A kinetic characterization of the glycerol released by 3 FeedBeads^®^ was performed under standard cultivation conditions, being 25°C and 180 rpm in an orbital shaker (Infors, Switzerland). The study was run per triplicate in 500 mL baffled shake-flasks with 50 mL buffered minimal media without carbon source (BM, 200 mM potassium phosphate, 1.34% YNB, pH 6 or pH 3). First, the effect of pH was evaluated by incubating three FeedBeads^®^ in BM shake-flasks at pH 6 and pH 3 for 72 h. Then, the kinetic characterization of 3 Feedbeads^®^ was performed in BM pH 6 by monitoring for 120 h the glycerol release under the same incubating conditions. Several samples were taken over time to determine glycerol concentration by HPLC.

### 2.2 RNA sequencing and analysis

A commercially available WT *K. phaffii* strain BSYBG10 (bisy GmbH, Hofstaetten/Raab, Austria) was cultivated in 25 mL minimal media (BM, 200 mM potassium phosphate pH 6, 1.34% YNB, 4 × 10^−5^% biotin) with 0.25% glycerol as carbon source in 250 mL baffled shake flasks for 9 h (28°C, 130 rpm, 80% humidity) until the initial glycerol was depleted. Afterwards, two glycerol FeedBeads^®^ (SMFB12001, Kuhner Shaker, Basel, Switzerland) were added, followed by the addition of 25 mL fresh minimal media supplemented with different inductors (xylitol, galactose, xylose, sorbitol) in duplicates to an end concentration of 0.25% in the media. In addition, one uninduced control culture was included as reference, only adding BM without any carbon source. After 3 hours, samples from the biological duplicates were taken and centrifuged at 5000xg. The cell pellet was frozen at −80°C and sent for RNA isolation and sequencing at BioGrammatics Inc. (Carlbad, CA, United States). Total RNA extraction, as well as quality control and library preparation were performed according to BioGrammatics standards. Poly-A enriched total RNA was sequenced as 50 bp single reads on an Illumina sequencer. Reads were mapped to the *K. phaffii* CBS7435 reference genome (Sturmberger et al., 2016) and read counts were generated using the STAR aligner (Dobin et al., 2013). Read and mapping quality was determined using fastqc (Andrews, 2010) and Qualimap (García-Alcalde et al., 2012). To compare expression strength between genes and samples, the transcripts per million (TPM) were calculated in the R/Bioconductor statistical environment (Gentleman et al., 2004; Robinson et al., 2010) using the edgeR package (Robinson et al., 2010). Genes were subsequently ordered by their median, mean and maximum TPM to identify highly expressed genes on all conditions.

### 2.3 Identification of the promoter sequence based on eGFP expression

To identify the promoter region, 1,000 bp upstream of the *HSP12* gene in *K. phaffii* were chosen to test for promoter activity and ordered as synthetic DNA at IDT (Coralville, IA, United States). Subsequently, 50 bp truncations were made down to 97 bp residual length to determine the minimal length of the promoter region capable to drive protein expression. Four versions were not clonable in first instance and were therefore discarded (PDH2, 9, 10, 17). The 14 cloned constructs consisted of the respective promoter, eGFP as reporter gene, the *AOX1* transcription terminator and a 1,100 bp homologous region to the 3′UTR of the *K. phaffii ARG4* gene, which was used to target the genome integration. The plasmid map and the truncated sequences can be found in the Supplementary File S1: Supplementary Figure S1; Supplementary Table S2, as well as transformation, cloning and cultivating protocol (Supplementary File S1). To facilitate integration into the right locus, the *K. phaffii* strain BSY11dKU70 was used (*aox1-/Mut* ^*s*^), which lacks the ability of non-homologous end-joining and therefore ensures targeted integration with over 90% efficiency (Näätsaari et al., 2012).

The screening cultures were performed in a semi-continuous manner on 96-deep well plate (DWP) format for 6 days while taking samples every 24 h for the first 4 days and a last sample after 132 h. Here, the fluorescence measured and normalized to the cell density as eGFP was expressed intracellularly. As a control the same construct was used with a constitutive promoter (P_*GAP*_) (Waterham et al., 1997) and a carbon source repressed promoter P_*CAT1*_ (Vogl et al., 2016) expressing also the eGFP. Cultivations were done in minimal media containing 1% glycerol or glucose as sole carbon source (BMG or BMD, 200 mM potassium phosphate pH 6, 1.34% YNB, 4 × 10^−5^% biotin and 1% glycerol or glucose).

### 2.4 CalB strain construction, screening and gene dosage determination

The parental strain BSYBG11 (*aox1-/Mut* ^*s*^), a single colony streak out of *K. phaffii* strain BG11 (BioGrammatics Inc.) deposited at bisy GmbH (Hofstaetten, Austria), was transformed with an expression vector containing the CalB gene under control of the P_DH_ promoter region. The procedure followed the same approach as Garrigós-Martínez et al. (2021). Briefly, the recombinant vector was based on pPpT4_Alplha_S vector. To avoid multi copy expression cassette integration, and thus also avoiding the gene dosage effect on the comparison, low amounts of linearized plasmid DNA (<1 µg of DNA) were used for transformation.

Several candidate clones were screened using glycerol as carbon source in DWP using BMG (200 mM potassium phosphate pH 6, 1.34% YNB, 4 × 10^−5^% biotin and 1% glycerol), as described by Garrigós-Martínez et al. (2021). Seven clones displaying average performance were selected and further rescreened in DWP in biological replicates, in order to validate the previous results. From these results, three clones were selected to check the gene copy number.

The gene copy/dosage number was determined using qPCR, following the method described by Abad et al. (2010). The protocol is described by Krainer et al. (2012) and based on the amplification of the Zeocin™ resistance-mediating gene Zeocin^®^, located on the integrated expression cassette, and the house-keeping gene *Arg4*. Therefore, the copy number of CalB gene was indirectly determined by the copy number verification of Zeocin^®^.

Accordingly, and including the producer clones previously constructed in the research group and described by Garrigós-Martínez et al. (2021), a set of 4 isogenic strains producing the CalB enzyme were used in the present study: GAP-C, PDF-C, UPP-C, and PDH-C. All the strains should only differ in the promoter used to regulate the CalB expression.

### 2.5 Shake-flask cultivations for CalB producer clones

The strains PDH-C, GAP-C, UPP-C and PDF-C were used for shake-flask cultivations, using 500 mL baffled flasks with 50 mL of buffered minimal glycerol media (BMG, 200 mM potassium phosphate pH 6, 1.34% YNB, 4 × 10^−5^% biotin and 1% glycerol). Shake-flasks were inoculated at an initial OD_600_ of 0.2 from an overnight pre-culture (growing in YPG (1% yeast extract, 2% peptone and 2% glycerol) at 25°C and 180 rpm). After 20 h of incubation at 25°C and 180 rpm, total glycerol depletion was confirmed by HPLC and three glycerol FeedBeads^®^ (SMFB12001, Kuhner Shaker, Basel, Switzerland) were added to start the feeding phase. The cultivations were run per triplicate and lasted for 72 h and several samples were taken over time for enzyme analysis.

The shake-flask cultures to test osmotic shock effect were carried out only with PDH-C strain at three different KCl concentrations: 0 M, 0.3 M, and 0.6 M. The cultivation protocol used was the same previously described in this section, but an osmotic shock was induced after 24 h by adding 10 mL of water, 2.4 M KCl or 3.6 M KCl to get 0 M, 0.3 M and 0.6 M KCl in the shake-flasks, respectively. Three FeedBeads^®^ were as well used for the feeding phase, running each osmolar condition per triplicate.

### 2.6 Fed-batch cultivations

All fed-batch cultivations were conducted in a 5 L *Biostat B* fermenter (Sartorius Stedim, Goettingen, Germany) starting with an initial volume of 2 L. The media composition was the same as described previosuly (Gasset et al., 2022), but with glycerol instead of glucose in the fed-batch feeding solution. For all cultivations, temperature was controlled at 25°C, pH permanently adjusted to 5 with NH_4_OH 15% v/v and the airflow was 2 L min^-1^. Dissolved oxygen (DO) was kept above 35% by stirring speed cascade (600–1,200 rpm) and by O_2_ enrichment if needed, always maintaining 2 L min^-1^ of total gas inlet flow. The software Eve^®^ (INFORS HT, Bottmingen, Switzerland) was used to monitor and control the cultivations.

#### 2.6.1 Fed-batch characterization

PDH-C, GAP-C and PDF-C strains were cultivated under carbon-limiting conditions at different specific growth rates (μ). After depletion of all glycerol contained in the batch media, an exponential pre-programmed feeding rate based on the biomass mass balance was started to maintain a constant μ (being 0.025 h^−1^ for PDH-C, 0.025 h ^1^ or 0.15 h ^1^ for GAP-C and 0.05 h^−1^ for PDF-C). All fermentations were stopped when the goal dry cell weight (DCW) of 80 g L^−1^ was achieved. More details about the fed-batch strategy are described in previous works of the group (Garcia-Ortega et al., 2013).

#### 2.6.2 Fed-batch optimization

Protein expression from the strain PDH-C was induced by pseudo-starving conditions, following two strategies: single pseudo-starving (PS) and three stages of pseudo-starving (3-PS). After the batch phase of the PS approach, a fast biomass growth fed-batch (constant μ of 0.15 h^−1^) was started to reach a goal biomass concentration of 70 g L^−1^ of dry cell weight (DCW). Then, a pseudo-starving phase was initiated by adding a constant glycerol feeding rate to reach the same overall specific glycerol uptake rate (q_S_) provided by three FeedBeads^®^ (0.005 g g_X_^−1^ h^−1^), which is equivalent to an initial µ of 0.003 h^−1^. In contrast, several cycles of growing—induction phases were performed in the 3-PS approach. Every biomass generating phase was conducted at a μ of 0.15 h^−1^, stopping at the target biomass concentration of 40, 60, and 80 g L^−1^ of DCW, which were followed by a 24 h period of the induction conditions. These induction conditions were designed to achieve a q_S_ of 0.005 g g_X_^−1^ h^−1^ with a constant feeding rate.

#### 2.6.3 Osmotic shock in fed-batch

Two fed-batch cultivations with PDH-C were performed using the PS approach and two different osmotic conditions: 0.3 and 0.6 M KCl. After the biomass generation phase, being around 70 g L^-1^ DCW, the appropriate volume of a 4 M KCl solution was added to achieve the target osmolarities of 0.3 and 0.6 M. Then, the constant feeding rate was started to reproduce PS conditions.

### 2.7 Biomass determination

For shake-flask cultivations, cell growth was monitored by measuring per triplicate the optical density at 600 nm (OD_600_) in a DR 3900 HACH Spectrophotometer (Hach Company, Ames, IA, United States). During fed-batch cultivations, biomass concentration was measured in quadruplicates in terms of dry cell weight (DCW), as described elsewhere (Cos et al., 2005). The relative standard deviation (RSD) in all measurements was lower than 5%.

### 2.8 Osmotic stress analyses

The actual osmolarity in the shake-flask and fed-batch supernatants of the osmotic shock studies were determined measuring the conductivity with a Cond 8 conductimeter (XS Instruments, Carpi, Italy).

### 2.9 Quantification of carbon source and by-products

HPLC (Dionex Ultimate 3,000, Dionex, Sunnyvale, CA, United States) with an ionic exchange column (ICSep ICE-COR- EGEL 87 H3, Transgenomic Inc., Omaha, NE, United States) was used to quantify the concentration of glycerol and other metabolites in the fed-batch cultures and the FeedBeads^®^ kinetic characterization samples. More details of the column and software used are described elsewhere (Jordà et al., 2014). RSD was below 1%.

### 2.10 Off-gas analyses

A *BlueInOne FERM* gas analyser (BlueSens, Herten, Germany) was used for monitoring CO_2_ and O_2_ molar fraction and absolute humidity from all fed-batches exhaust gas. To get more accurate measurements, the gas analyzer was re-calibrated in each fed-batch. The data recorded were used to calculate key respirometric parameters: oxygen uptake rate (OUR), carbon dioxide evolution rate (CER), their corresponding specific rates (qO_2_ and qCO_2_) and respiratory quotient (RQ). RSD was less than 5% in all cases.

### 2.11 Enzymatic analyses

Lipolytic activity of secreted CalB was determined using a p-nitrophenyl butyrate (pNPB)-based assay (2635–84–9, Merck, Darmstadt, Germany). The reaction buffer was as follows: 300 mM Tris-HCl pH 7 containing 4.83 mM p-NPB and 0.93% acetone. After pre-heating the buffer at 30°C, 900 μL of reaction buffer were mixed with 100 μL of culture supernatant. P-nitrophenol color development at 405 nm was monitored in a Specord 200 Plus spectrophotometer (Analytik Jena GmbH, Jena, Germany) at 30°C for 2 min. One activity unit was defined as the amount of enzyme needed to release 1 μmol p-nitrophenol · min^-1^ under assay conditions. RSD was below 4%.

Further protein analyses of fed-batch supernatant samples were performed by SDS-PAGE. For sample preparation, 15 µL of supernatant were mixed with 5 µL of loading buffer (Laemmli buffer 4x + β-mercaptoethanol in 10:1 relation, Biorad, Hercules, CA, USA) and incubated at 95°C for 5 min. After cooling down, 15 µL were loaded into 4%–15% Mini-PROTEAN TGX Precast Protein Gels (Biorad, Hercules, CA, United States) and run at 120 V for about 80 min. The visualization and analyses of the gel was performed using Image Lab (Biorad, Hercules, CA, United States).

### 2.12 Consistency test and data reconciliation

The biomass elemental composition of *K. phaffii* growing on glycerol at different growth rates was previously determined by Tomàs-Gamisans et al. (2018). For fed-batch characterization, yields and rates were calculated using equation based on mass balances, that can be found elsewhere (Garcia-Ortega et al., 2016; Ponte et al., 2016). In all cases, carbon and electron data recovery was above 90%. To further validate the results, measurement consistency was checked and reconciliation procedures were applied, constraining by carbon and electron balances (Ponte et al., 2016). The confidence level reached in the statistical consistency test was 95% for all fed-batch characterization.

## 3 Results and discussion

### 3.1 FeedBeads^®^ release kinetic characterization

When developing and implementing new process strategies for RPP, a significant operational gap still exists between early-stage cultivations in shake-flask or DWP and fed-batch processes in fermenters. However, working under process-relevant conditions is crucial when performing small scale cultivations (Looser et al., 2015). The so-called FeedBeads^®^ developed by Kuhner-shaker GmbH (Germany) would bridge this gap, since shake-flask cultivations can be fed under fed-batch-like conditions. To study the conditions this technology provides, a characterization of glycerol release FeedBeads^®^ was carried out in shake-flasks at standard cultivation conditions.

Based on the product specifications, three FeedBeads^®^ were used to characterize the release kinetic. First, since *K. phaffii* acidifies the media when growing, the glycerol release was tested at pH 6 (buffered minimal media standard) and 3 (acidic pH) at 25 °C. No differences were detected after 72 h of releasing (data not shown) between the two pH values, suggesting that the kinetic is not pH-dependent.

**FIGURE 1:**
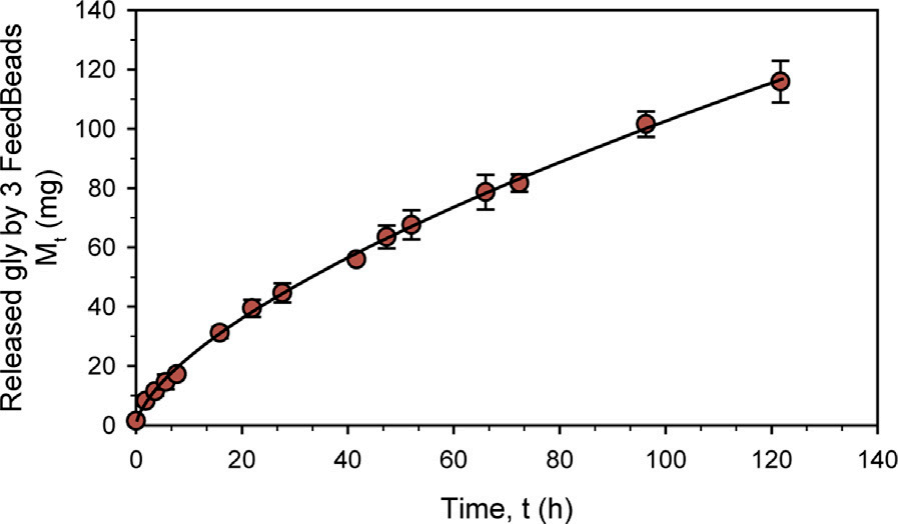
Characterization in shake-flask of three FeedBeads^®^ releasing glycerol at 25°C in BM. The dots indicate the released glycerol amount measured by HPLC, while the line describes the release kinetic. The error bars indicate the standard deviation between triplicates.

In order to characterize glycerol release kinetic, a new experiment was performed with BM at pH 6°C and 25 °C for 120 h. As can be seen in Figure 1, glycerol release followed a non-linear kinetic typical profile drug release. Ritger and Peppas (Ritger and Peppas, 1987) developed a simple relationship that describes the solute release from discs, which was adapted to:

$$M_{t}=kt^{n}$$

where M_t_ is the amount of solute released (mg), t is the release time in hours, k is a constant and n is the diffusional exponent, which is characteristic of the release mechanism. Using this formula, the kinetic model obtained to explain the mg of glycerol released by 3 FeedBeads^®^ over hours was:

$$M_{t}=5.13t^{0.65}$$

which fully describes the release kinetic with an r^2^ of 0.999 and a standard error of 1.132 (Figure 1).

According to the diffusional exponent (0.65), the FeedBeads^®^ release mechanism is due to diffusion and erosion of the matrix (Padmaa Paarakh et al., 2018). The glycerol release kinetic determined for the glycerol FeedBeads^®^ was in the same order as previously found for the glucose FeedBeads^®^, being 1,34 t^0,77^ and 1,63 t^0,74^ mg of glucose per hour and one FeedBeads^®^ (Prielhofer et al., 2015; Halka et al., 2018).

Taking this glycerol release kinetic, the growth parameters of a standard *K. phaffii* shake-flask cultivation can be predicted assuming that all the glycerol released by the FeedBeads^®^ is immediately consumed. Therefore, when adding 3 FeedBeads^®^ after a batch phase conducted with 1% BMG, the specific growth rate (µ) would be 0.003 h^-1^ and the specific substrate uptake rate (q_s_) 0.005 g_S_ g_X_^−1^ h^−1^. Accordingly to the usual µ used in fed-batch cultures, which is in the range from 0.025 h^-1^ to 0.15 h^-1^, the use of 3 FeedBeads^®^ would allow severe carbon-limiting conditions.

### 3.2 Identification of a novel promoter based on RNA sequencing

In order to identify a novel promoter by seeking highly expressed genes under specific conditions, the use of RNA-seq was considered more suitable than microarrays since it offers a higher dynamic range. Furthermore, RNA-seq is not restricted to known genes and the resulting gene expression measures are highly reproducible (Nagalakshmi et al., 2008). Therefore, with the aim of finding a methanol-free and tightly regulated promoter under glycerol restriction, RNA from a WT *K. phaffii* cultivation under glycerol de-repressed conditions was isolated to perform RNAseq analyses (The transcriptome data was made available under ENA project PRJEB58889).

**FIGURE 2:**
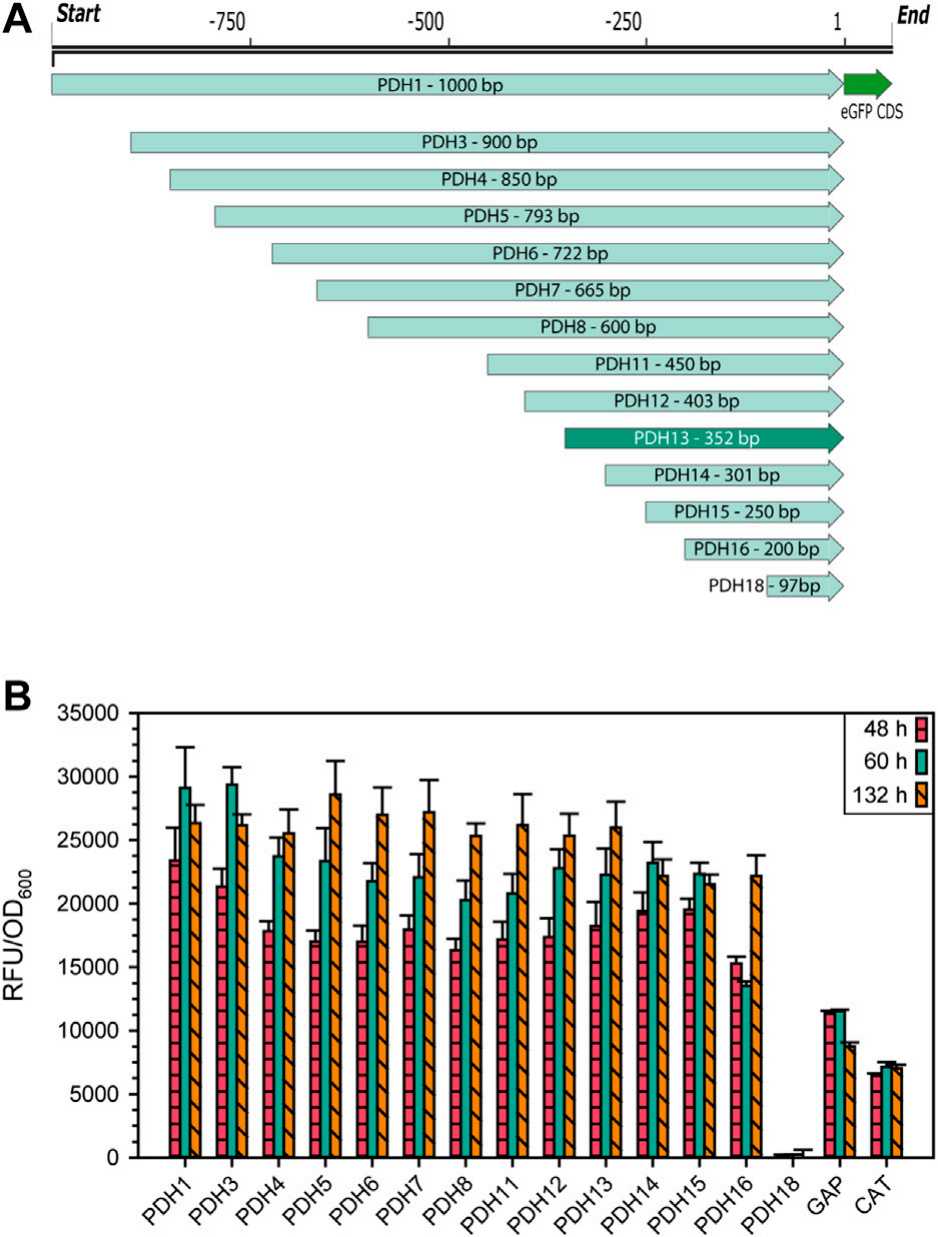
**(A)** Schematic representation of 14 PDH promoter truncations for identification of the essential promoter region with the reporter protein eGFP. **(B)** Expression levels of the different PDH promoter truncations in raw fluorescence units normalized by cell density. Cells were cultivated in biological 7-fold replicates which represent the standard deviation, taking samples after 48, 60, and 132 h of cultivating with glycerol as sole carbon source. As controls, the same construct was used with P_*GAP*_ and P_DC_ instead of P_DH_.

The gene that showed the highest mean, median as well as maximum transcripts per million (TPM), was the *ACIB2EUKG772368*, a homologue to the heat shock protein 9/12 from *S. cerevisiae* (Supplementary File S2: Supplementary Table S4), hereafter called *HSP12* in accordance with its homologue in *S. cerevisiae*. As this gene was highly expressed in all the tested conditions and was previously reported to be highly expressed under stress conditions, its promoter was considered an interesting candidate to develop an alternative expression system based on it. To examine the promoter region, 1,000 bp upstream of the start codon were ordered as synthetic DNA. Systematic truncations on the 5′ end were made by PCR, while also adding appropriate overhangs to the eGFP expression vector for Gibson Cloning (Gibson et al., 2009). An overview of all truncations is shown in Figure 2A. As reporter gene, the intracellularly expressed fluorescence protein eGFP was used. The vector also contained 5′ and 3’ homologous regions to the *ARG4* locus in *K. phaffii* to minimize ectopic genomic integration and make the results more comparable. The eGFP expression levels obtained growing on glycerol (Figure 2B) and glucose (Supplementary File S1: Supplementary Figure S4) were rather similar. The shortest variant of the putative promoter sequence still showing the same expression level as PDH1 (1,000 bp) was PDH13 (Figure 2B) with only 352 bp, whose promoter sequence will be referred for now on as P_DH_.

### 3.3 Strain generation, screening, and gene dosage determination

To study the performance of the novel isolated P_DH_ promoter controlling CalB expression and compare the results with other new expression systems previously reported by Garrigós-Martínez et al. (2021), the same cloning approach was followed to generate an isogenic P_DH_-based CalB producer clone. Due to the high clonal variability that *K. phaffii* presents, one of the critical steps in bioprocess development is selecting an appropriate producing clone (Looser et al., 2015). Since the aim was to isolate a clone with a single expression cassette integrated in the genome, a high-throughput screen of 32 clones was performed in DWP using glycerol as sole carbon source, with the scope of selecting an averagely producing clone. Seven putative single-copy integration transformants were rescreened in biological triplicates and the previous results were validated. The single cassette integration of three of these candidate clones was determined by qPCR (Supplementary File S1: Supplementary Tables S2, S3). Therefore, a confirmed single copy clone with an average CalB production was selected for characterizing its regulation and performance, being named PDH-C.

### 3.4 Shake-flask cultivations, first insights into PDH-C system

Since the regulation of *HSP12* in *S. cerevisiae* is related to stress damage, temperature and pH stressing conditions were applied to preliminary deep-well plate cultivations with PDH-C to induce CalB expression (Praekelt and Meacock, 1990). However, no positive or inducing effect was observed compared to standard cultivation conditions (unpublished data). As reported in *S. cerevisiae*, *HSP12* is repressed in the exponential phase and activated when the cells enter stationary phase (de Groot et al., 2000). Therefore, new culture strategies were designed with the aim to validate the potential of P_DH_ for controlling the target recombinant expression in growth decoupled conditions. To do so, PDH-C was cultivated in shake-flask until total glycerol depletion was confirmed. Then, three glycerol FeedBeads^®^ were added to reach the desired stationary state by applying severe carbon-limiting conditions. The benchmark strains GAP-C, UPP-C and PDF-C were cultivated under the same conditions.

**FIGURE 3:**
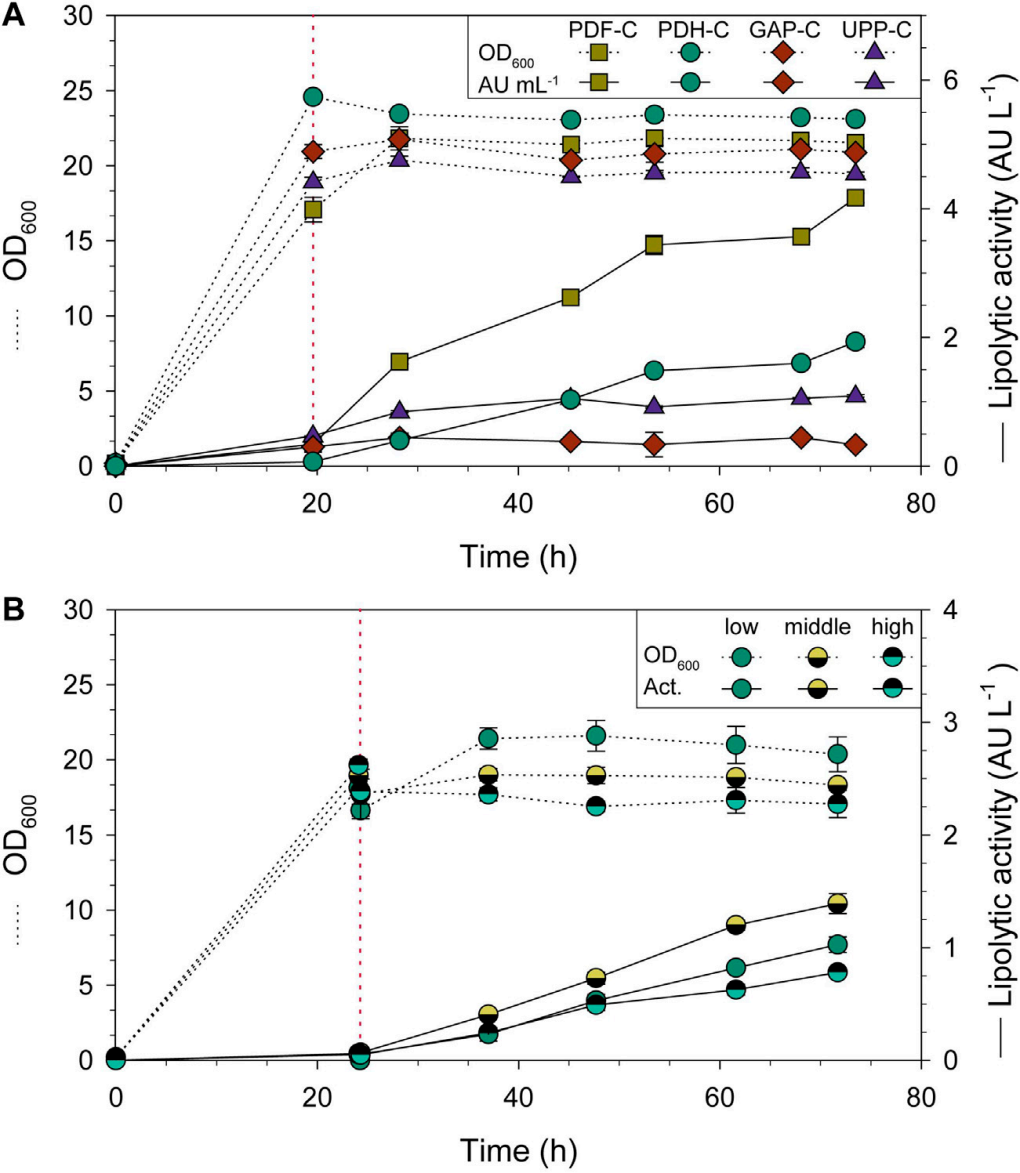
Shake-flask cultivations of CalB producing strains using three glycerol FeedBeads^®^ (addition time is indicated by red dotted lines). Time-course evolution of OD_600_ (dotted line) and lipolytic activity (solid line). **(A)** PDH-C performance is compared to the benchmarks GAP-C, UPP-C and PDF-C. **(B)** Omotic shock cultivations of PDH-C at low, middle and high osmolarities. The red line also indicates the 10 mL addition of water or KCl solution. The error bars indicate the standard deviation between biological triplicates.

For all the strains compared, the biomass in terms OD_600_ was rather constant after the addition of FeedBeads^®^ (Figure 3A). The average specific growth rate (µ) during the feeding phase was around 0.003 h^-1^ for all strains, while the average specific substrate uptake rate (q_s_) was 0.005 g_S_ g_X_^−1^ h^−1^. Regarding CalB production by PDH-C (Figure 3A), almost no lipolytic activity was detected in the supernatant when cells were growing exponentially in carbon-excess conditions (batch phase). In contrast, CalB production rose sharply when adding the FeedBeads^®^, achieving 5.2-fold and 1.5-fold more lipolytic activity (normalized by OD_600_) than GAP-C and UPP-C, respectively. PDF-C instead, showed 2.3-fold more lipolytic activity than PDH-C.

The shake-flask results confirmed that three FeedBeads^®^ provided enough glycerol for cell maintenance and CalB production, but not for growth, achieving the desired stationary phase conditions from now on called pseudo-starving. In PDH-C, these conditions allowed to induce CalB production, suggesting that P_DH_ is tightly repressed by glycerol, observing no induction in the growing phase. These results are coherent with the previously published studies in *S. cerevisiae* using glucose as carbon source (de Groot et al., 2000). Moreover, after addition of the FeedBeads^®^ the cultivation entered the so-called pseudo-starving condition in which the performance of PDH-C even surpassed the performance of the constitutive promoter-based UPP-C and GAP-C. CalB activity in UPP-C and GAP-C supernatants was virtually constant after the addition of FeedBeads^®^, since both are growth-related expression systems (Garrigós-Martínez et al., 2021). In contrast, for PDF-C containing the strong and tunable promoter P_DF_ (Garrigós-Martínez et al., 2021), the lipolytic activity in the supernatant increased markedly over time, outperforming PDH-C in terms of CalB production (Figure 3A). Despite this, the P_DH_-based expression system was considered valuable for further studies in more scalable cultures, such as bioreactors, due to its non-growth-coupled performance.

As recently published, *HSP12* belongs to a protein family whose role is to repair stress-related damage, being one of the mediators of hyperosmotic stress adaptation in *K. phaffii* (Wang et al., 2020). With the aim to further enhance the production performance, new shake-flask cultivations with the PDH-C were carried out at different osmolarities to induce osmotic stress.

The desired osmolarities were reached by adding the same volume of different concentrations of KCl solution. This osmolyte was chosen since it was already present in the bioreactor culture medium. Cell growth was clearly affected when KCl was added at a concentration of 0.4 M, 0.6 M and 1.2 M at the beginning of the cultivation, with the maximum growth rate (µ_max_) markedly reduced at the highest osmolarity (data not shown). Therefore, it was decided to not proceed experiments with a KCl concentration of 1.2 M and add a pulse of water or KCl to each cultivation after the batch phase, to get a final KCl concentration of 0 M, 0.3 M and 0.6 M. These concentrations resulted in a cultivation osmolarity of 24.4, 59.2, and 88.4 mS/cm, respectively, which will be named for now on low, middle and high osmolarity (Dragosits et al., 2010).

After the pulses and FeedBeads^®^ addition the OD_600_ was again rather constant (Figure 3B). As expected, CalB production was triggered just after starting the induction phase for all three cultivations (Figure 3B). Compared with low osmolarity, a 35% increase in lipolytic activity was achieved at middle osmolarity, while a decrease of 25% was observed for high osmolarity. According to Dragosits et al., 2010, high osmolarity upregulates the unfolded-protein response (UPR) and the expression of general stress related proteins in WT *K. phaffii* strains. Nevertheless, in the cited article these changes were lower and even undetectable in the producing strain (a recombinant antibody fragment under P_*GAP*_ control), since the stress responses were already induced due to protein over-expression. When growing PDH-C under middle and high osmolarity conditions, it could be hypothesized that P_DH_ was already close to being fully activated by pseudo-starving conditions and protein over-expression, which might explain the low CalB increase and decrease after osmotic shock. Nonetheless, since it was considered interesting to study these cultivation conditions in a more controlled environment such as a bioreactor, further fed-batch cultures were also performed and reported in the present work.

### 3.5 Fed-batch characterization in carbon-limiting conditions

Currently, fed-batch cultivation is the most widely used operational mode for RPP, since it allows to reach higher cell densities and product titers in a controlled environment. More specifically, a pseudo-stationary state is achieved when implementing an exponential feeding profile that controls and maintains a constant µ, working under carbon-limiting conditions (García-Ortega et al., 2019). To characterize PDH-C performance under these controlled conditions and according to the shake-flask results, a fed-batch cultivation was carried out at a low constant µ of 0.025 h^−1^. As a benchmark, two fed-batch cultivations at high (0.15 h^−1^) and low (0.025 h^−1^) µ were performed with GAP-C, considering the high µ cultivation as the optimal strategy for P_*GAP*_ in terms of productivity (Nieto-Taype et al., 2020).

**FIGURE 4:**
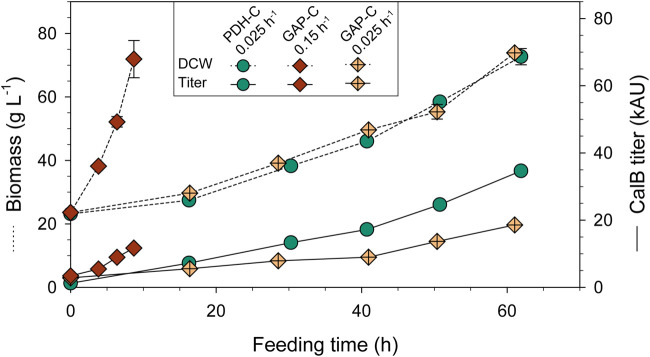
Biomass and CalB titer time-course of carbon-limiting fed-batch cultivations with PDH-C at low µ (0.025 h^-1^) and GAP-C at high (0.15 h^-1^) and low µ (0.025 h^-1^). The errors bars represent the standard deviation of the measurements.

As expected, biomass production increased exponentially over time and in agreement with the set-point µ (Figure 4), reaching a DCW of almost 80 g L^−1^ in all fed-batch cultivations. As can be observed in Table 1, GAP-C at low μ and PDH-C presented the same q_S_, while GAP-C at high μ had a greater one in agreement with the target μ. Moreover, all key parameters that are related to the physiological state of the cultivation were consistent with the ones obtained in chemostat cultivations performed with GAP-C and the other isogenic clones producing CalB, such as PDF-C and UPP-C (Garrigós-Martínez et al., 2021). These similarities suggested that CalB production with the new promoter P_DH_ did not alter the physiological state of the cultivation.

**TABLE 1:** Physiological and product-related parameters from carbon-limiting fed-batch cultivations with PDH-C at low µ (0.025 h^-1^) and GAP-C at high (0.15 h^-1^) and low µ (0.025 h^-1^). All parameters are calculated from the feeding phase.

	GAP-C 0.025	GAP-C 0.15	PDH-C 0.025
Real μ (h^−1^)	0.025	0.15	0.025
q_s_ (g_S_ g_x_^−1^ h^−1^)	0.046	0.25	0.044
Y_x/s_ (g_x_^−1^ g_S_^−1^)	0.54	0.61	0.58
RQ	0.72	0.68	0.70
Titer (kAU)	18.58	11.70	26.82
Y_p/x_ (AU g_x_^−1^)	100.45	61.28	185.22
q_p_ (AU g_x_^−1^ h^-1^)	2.45	9.44	4.59

In relation to recombinant protein production, CalB levels increased exponentially over time from the very beginning of the culture for both GAP-C cultivations, due to the growth-coupled nature of the expression system (Türkanoğlu Özçelik et al., 2019). In contrast, CalB titer in PDH-C was almost negligible after the batch phase but rapidly increased in the feeding phase following a non-exponential profile. At the end of the cultivations, PDH-C at low μ surpassed GAP-C at low and high μ, presenting 1.4- and 2.3-fold titer increases, respectively. Nevertheless, GAP-C cultivation at high µ presented 2.1-fold higher specific production rate (q_p_) than PDH-C at low µ due to the shorter cultivation time (Table 1). On the other hand, in terms of operational requirements this higher-growth GAP-C cultivation should be considered less advantageous in comparison with PDH-C cultivation performed at low µ, since the faster the growth rate the higher the cooling and oxygen demands. For both mentioned cases, however, those operational requirements would still be far from the demanding methanol-based bioprocesses. On balance, although PDH-C results were promising as starting point, the enhancement in CalB production in relation to GAP-C was much lower than in shake-flasks cultivations, suggesting that there was room for improvement in the operational strategy.

### 3.6 Fed-batch optimization by pseudo-starving

When RPP bioprocess is optimized, the usual Key Performance Indicators (KPI) to maximize are the product to biomass yield (Y_P/S_), the volumetric productivity (Q_P_) and the final product titer. Prioritizing one or another ultimately will always depend on the product application. Most certainly, biomass amount will greatly affect the production, but considering that in the end it is usually a waste by-product, the maximum amount reachable should be limited into range of between 80–100 g L^-1^ DCW (García-Ortega et al., 2019). Accordingly, a PDH-C fed-batch optimization process was carried out by mimicking the pseudo-starving conditions achieved in shake-flask cultivations, performing a single induction phase (PS) or three at different biomass concentrations (3-PS). For all the strategies, the mentioned biomass concentration limit was never exceeded. The induction through pseudo-starving was implemented by setting a constant glycerol feeding rate to achieve a q_s_ of 0.005 g_S_ g_X_^−1^ h^−1^, which means an initial μ of 0.003 h^-1^.

**FIGURE 5:**
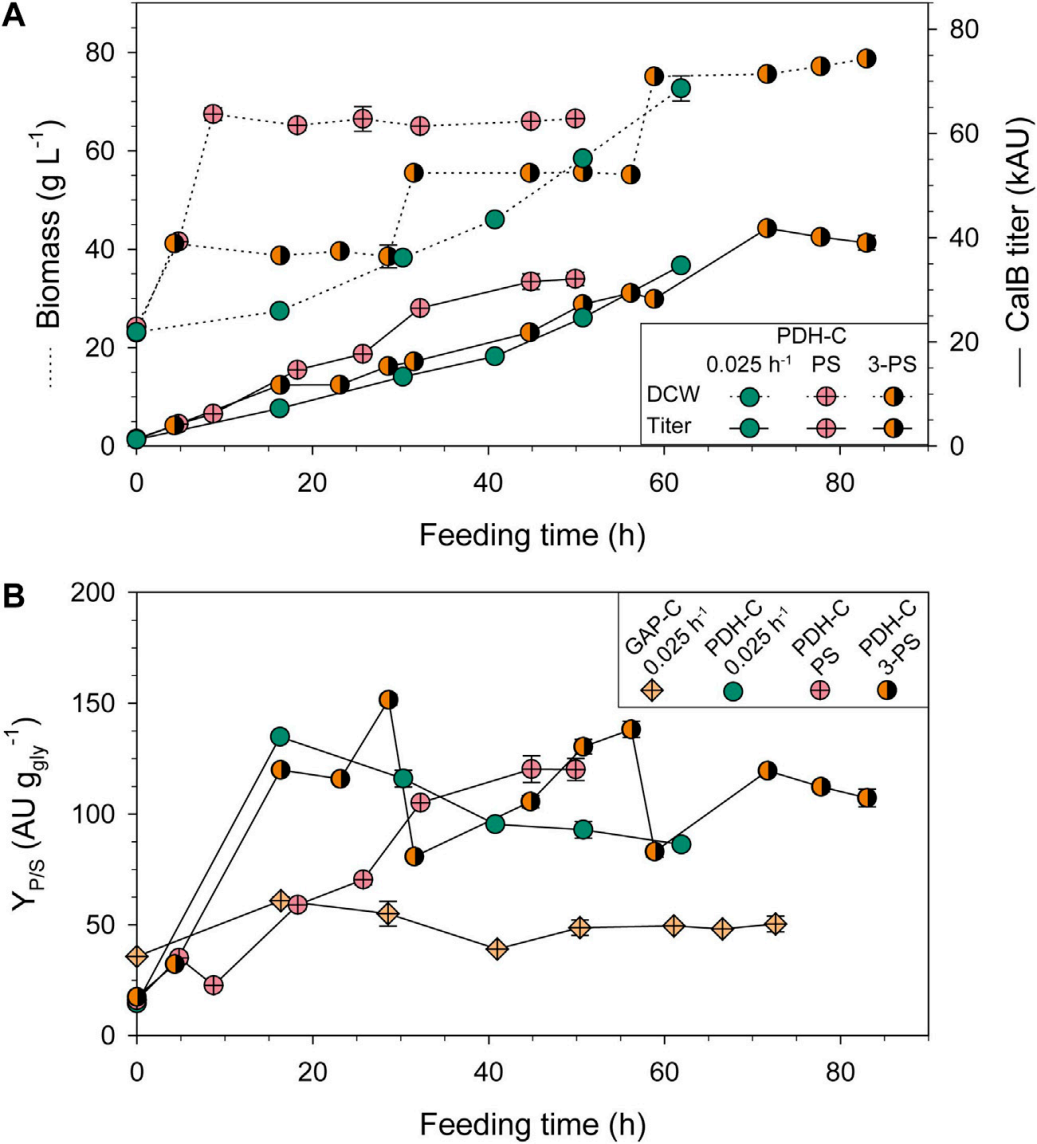
Optimization of PDH-C fed-batch cultivations through pseudo-starving conditions compared to the benchmark cultivation of GAP-C at low µ (0.025 h^-1^). **(A)** Biomass and CalB titer evolution over time of PDH-C optimized cultivations (PS and 3-PS) and at low µ (0.025 h^-1^). **(B)** Product to substrate yield (Y_P/S_) of the PDH-C optimized cultivations compared to PDH-C and GAP-C at low µ (0.025 h^-1^). The errors bars represent the standard deviation of the measurements.

The PDH-C biomass and CalB production profiles conducting PS and 3-PS approaches are compared to PDH-C fed-batch at low µ (FB-0.025) in Figure 5A. In terms of cell growth, PS and 3-PS kept the biomass constant around the selected DCW in the induction phases, following the desired profile observed in shake-flasks when adding the FeedBeads^®^. As expected, in the PS approach CalB production slightly increased as the biomass grew but boosted when the induction was applied, verifying that pseudo-starving conditions were successfully implemented in this approach. Interestingly, during the first 24 h of induction the production increased rather linearly over time, reaching a similar titer as PDH-0.025 at the same DCW. After that, the curve started to flatten similar to the behavior observed in shake-flasks. *K. phaffii* has been demonstrated to have a better adaptation to severe carbon starvation conditions than *S. cerevisiae*, showing a high viability and low maintenance requirements at near-zero specific growth rates (Rebnegger et al., 2016). During all PS cultivation, high cell viability was observed according to the physiological parameters and the lack of visible cell lysis in SDS-PAGE (Supplementary File S1: Supplementary Figure S2), indicating that the stop of CalB production at the end of the cultivation was not caused by cell lysis. Additionally, the absence of lower molecular weight bands on SDS-PAGE, which would indicate CalB proteolysis, suggests that no active host proteases were present. Moreover, this dynamic expression was also observed in *S. cerevisiae* after applying different stressing conditions to activate P_*hsp12*_, being followed by GFP fluorescence (Karreman and Lindsey, 2005; Xiong et al., 2018) and *HSP12* relative transcript levels (Chowdhary et al., 2017; Ren et al., 2022). In accordance, P_DH_ activation in *K. phaffii* also seemed to decrease over time, although the stressing conditions were maintained.

These observations inspired the 3-PS design, in which we reduced the induction phases to 24 h and then reset the likely non-producing state of the cultivation by starting a new biomass production phase. As a result, the CalB titer successfully increased almost linearly in the first two induction phases of the 3-PS cultivation. However, during the third induction phase the production ceased after 12 h. Although the PS-3 cultivation achieved 1.3-fold more titer than the PS cultivation, the investment of induction time was 2.5-fold higher, making the bioprocess more complex and time-demanding. Therefore, PS was the strategy chosen for further experiments.

### 3.7 Fed-batch optimization by osmotic shock

With the goal to further boost the RPP using the P_DH_-based expression system, also osmotic shock was applied to the PS approach to increase P_DH_ activation. Again, shake-flask conditions were reproduced in the bioreactor, performing a pulse of KCl solution at the beginning of the induction phase to get a middle and high osmolarity of 0.3 and 0.6 M, respectively. The conductivities measured during the induction phase was 45.5 and 82.4 mS/cm, respectively, which were in line with measurements of shake-flask cultivations. The results of PS fed-batch cultivation at middle (PS-middle) and high (PS-high) osmolarity were compared to the standard PS cultivation (16.5 mS/cm), in terms of total biomass and CalB produced.

**FIGURE 6:**
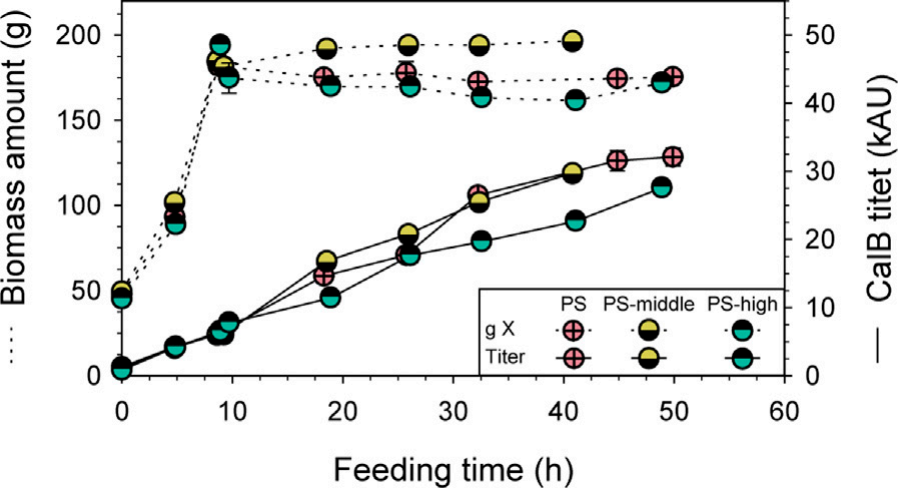
Optimization of PDH-C fed-batch cultivations through osmotic shock compared to the benchmark cultivation of PDH-C PS. Biomass and CalB titer evolution over time are represented of PDH-C cultivations at high, middle and low (PS) osmolarity. The errors bars represent the standard deviation of the measurements.

Practically, no differences were observed in total biomass and CalB titer during the biomass production phase of all three fed-batch cultivations (Figure 6), exhibiting the high reproducibility of the platform used. Unfortunately, no remarkable differences in CalB titer were observed between PS and PS-middle cultivations, while the performance of PS-high cultivation suggested that the high osmolarity caused a detrimental effect. In fact, when the pulse of KCL solution was added to PS-high cultivation, a slight decrease in total biomass was observed (10%) and a markedly lower final CalB production was achieved. However, no evident cell lyses in PS-high cultivation was detected by SDS-PAGE (Supplementary File S1: Supplementary Figure S3) although the foaming observed right after the KCl solution pulse. Moreover, the lower lipolytic activity of PS-high cultivation was consistent with protein band intensity in the SDS-PAGE.

The improvement in CalB production observed in shake-flask scale when cultivating PDH-C at middle osmolarity could not be reproduced in a bench-top bioreactor, since none of the osmolarity strategies tested could further boost the target RPP. Therefore, applying two stresses at the same time, pseudo-starving and osmotic shock, apparently did not have a synergetic effect for the P_DH_ activation.

### 3.8 PDH-C, a promising platform for growth-decoupled protein expression approaches

Considering the scalability of the fed-batch process and from an operational point of view, one of the most important features to minimize are heat production and O_2_ consumption. While the KPI to maximize are usually the productivities and/or the final titer, depending on the market of the target protein. For PDH-C, the best optimization approach for high productivities was PS cultivation, since it allowed to achieve the highest specific (Q_p_) and volumetric (Q_v_) productivity (Table 2) while keeping the process relatively simple. On the other hand, 3-PS cultivation achieved the highest final titer comparing to all GAP-C and PDH-C cultivations, which meant 2.3 times more than the best GAP-C production performance. This parameter is critical for high value-added products since high titers allow to reduce the downstream processing costs (García-Ortega et al., 2019). Comparing the achieved Q_p_ with the benchmarks, PS cultivation showed a 3.6-fold increase and a 3.2-fold decrease in relation to GAP-C 0.025 and PDF-C 0.05, respectively. Regarding GAP-C 0.15, the final titer was 2.3-fold higher in PS cultivation while Q_p_ was quite similar. Furthermore, regarding the osmotic shock approaches, PS-high and PS-middle cultivations showed lower Q_p_ in reference to PS, as expected. All these Q_p_ differences are also applicable to Q_v_, although the final biomass was slightly different in each process.

**TABLE 2:** Comparison of key performance indicators of PDH-C standard and optimized fed-batch cultivations with the benchmarks GAP-C and PDF-C fed-batch cultivations. All the parameters are calculated from the feeding phase.

	GAP-C 0.025	GAP-C 0.15	PDH-C 0.025	PDH-C PS	PDH-C 3-PS	PDF-C 0.05	PDH-C PS-middle	PDH-C PS-high
Real μ (h^−1^)	0.025	0.15	0.025	-	-	0.052	-	-
Specific productivity (AU g_X_^−1^ h^−1^)	1.25	5.03	2.09	4.53	2.68	14.42	3.85	3.37
Volumetric productivity (AU L^−1^ h^−1^)	92.67	361.44	151.66	294.21	202.78	1,056.39	272.29	182.32
Y_p/s_ (AU g_S_^−1^)	49.54	35.55	84.03	105.11	119.54	298.81	94.13	72.36
Titer (kAU)	18.58	11.46	26.82	26.48	41.82	90.67	25.52	19.65
Lipolytic activity (AU mL^−1^)	6.69	4.35	9.82	9.97	15.04	33.36	9.17	6.19

In general, using glycerol as a carbon source is economically more attractive than glucose or methanol. Moreover, crude glycerol from biodiesel industry can be used for *K. phaffii* fermentations with no extra refinements needed, promoting the circular bioeconomy (Potvin et al., 2012; Robert et al., 2017). Beside using a cheaper carbon-source, reducing the amounts needed for the process can have a great impact on the production costs. Therefore, the product to substrate yield (Y_P/S_) is usually also a crucial parameter to maximize, especially for low added-value products. In PDH-C PS cultivation strategy, the overall Y_P/S_ was 2.1-fold higher and 2.8-fold lower than GAP-C 0.025 and PDF-C 0.05, respectively (Table 2). Interestingly, the growth-coupled regulation of GAP-C was clearly observed in Figure 5B, where the evolution of the accumulated Y_P/S_ over time was kept constant. In contrast, the linear trend in PDH-C showed different slopes depending on the feeding strategy applied (Figure 5B). When starting the fed-batch phase at a constant µ of 0.025 h^−1^ (PDH-C 0.025), a marked increase in Y_P/S_ was observed in the first 16 h, which then decreased linearly over time. In PDH-C PS, the stress by pseudo-starving was initiated once the biomass was grown to 70 g L^−1^, which resulted in a Y_P/S_ linear increase for 30 h before starting to saturate. Since this saturation was already observed in CalB titer, the pseudo-starving application in PDH-C 3-PS was performed for 24 h at 40, 60, and 80 g L^−1^ of DCW. As in CalB titer, the Y_P/S_ increased linearly over time in each induction phase except for the third one, which stopped after 12 h of induction.

The slopes achieved in the different induction phases of PS and 3-PS suggested that the lower the biomass is, the higher CalB is produced. This statement would agree with the high activation observed in PDH-C 0.025 when the biomass was 22 g L^−1^, activation that did not stand over time since the cultivation was actually growing. However, the shift from growing at µ_max_ to 0.025 h^−1^ was most likely enough to activate P_DH_ at the beginning. Therefore, this process parameter not only shows the growth-decoupled regulation of P_DH_ and its transient activation, but also the impact that the initial biomass can have. Although biomass can be used as single-cell protein (SCP) for food industry (Jach et al., 2022), working at low cell densities but achieving high titers makes P_DH_-based expression system more advantageous for industrial scale bioprocess, since problems related to mixing time could be minimized (Junker, 2004).

Based on the results of all PDH-C cultivations, one could theorize that this expression system is highly repressed in the presence of glycerol. In *S. cerevisiae* though, there has been evidence that glucose-6-phosphate formation is essential for *HSP12* repression, however the signal transmission pathway is still unknown (de Groot et al., 2000). When growing *K. phaffii* in chemostat cultivations with glycerol as a carbon source, an increase in the metabolic flux distribution through gluconeogenesis, and therefore glucose-6-phosphate formation, was detected as the μ increased (Tomàs-Gamisans et al., 2019). Although really low μ were not studied, it is quite plausible that P_DH_ regulation also depends on glucose-6-phosphate formation, which might be inexistent at pseudo-starving conditions. Consequently, this regulation provides not only a growth-decoupled expression system, but also a slightly tunable one depending on the μ selected.

Despite the promising results described, the heterologous P_DF_ is still the strongest promoter reported in *K. phaffii* so far, presenting a growth-dependent regulation with its maximum at medium µ (Vogl et al., 2020; Garrigós-Martínez et al., 2021). Nevertheless, both expression systems showed a similar protein quality with no CalB degradation (Supplementary File S1: Supplementary Figure S2). Non-etheless, P_DH_ can tightly repress the recombinant expression during biomass growth. This regulation represents an advantage over P_DF,_ since protein folding might be facilitated due to the low metabolic burden that protein overexpression and cell maintenance can cause to the cell machinery (Wu et al., 2012). Besides, having detached growth and production phases represents a better choice to produce toxic proteins that have detrimental effects on growth. This is the case of the widely studied *Rizhopus oryzae* lipase, which cannot be constitutively expressed but has been efficiently produced in methanol-induced expressions systems (López-Fernández et al., 2021).

As an example of this growth-production detachment, while GAP-C and PDF-C presented respectively the 15.4% and 7.7% of the total final CalB activity at the end of the batch phase, PDH-C produced only the 4.8%. Moreover, some high-value added products require a moderate expression to produce functional proteins that meet the pharmaceutical grade (Juturu and Wu, 2018). Therefore, although P_DH_ is not as strong as P_DF_, its growth-decoupled regulation might enable a more efficient production of certain recombinant proteins, while still implementing a methanol-independent approach. In fact, this turn-on/off regulation controlled by simply minimizing the glycerol feeding rate must be especially highlighted, since it represents an operational advantage over carbon-controlled inducible promoters that require a transition phase. Consequently, P_DH_ expands the *Pichia* toolbox to efficiently produce recombinant proteins, an alternative option especially for the low added-value products that are essential to promote the transition towards a circular bioeconomy.

## 4 Conclusion

The more popular *K. phaffii* becomes as a cell factory for RPP the more expression systems with a diverse regulation are required by the industry, which is currently critical for establishing a circular bioeconomy. Accordingly, in this study a methanol-free and growth-decoupled expression system based on the new promoter P_DH_ has been identified and studied under process-relevant conditions, from shake-flask to benchtop fed-batch cultivations. P_DH_ was identified as the main regulatory sequence of the endogenous *HSP12* gene, which presumably codes for heat-shock protein 12. From the results of this study, it can be stated that P_DH_ is highly and transiently induced upon carbon source limitation, the so-called pseudo-starving conditions, which corresponds well to the native function of HSP12 in *S. cerevisiae* (de Groot et al., 2000). Considering that under almost total carbon-source starvation, the CalB expression is transiently boosted, we hypothesize that glucose-6-phosphate formation, a metabolite whose flux is reduced at lower growth rates, is essential to tightly repress P_DH_ *.*

In shake-flask cultivations, pseudo-starving conditions were achieved by employing the feeding technology FeedBeads^®^, which slowly release a defined amount of glycerol over time. Using this strategy, PDH-C surpassed the benchmark producing clones GAP-C and UPP-C, but not PDF-C. To further investigate the potential of PDH-C with the aim to optimize the process, a set of fed-batch cultivations were successfully conducted under standard carbon-limiting conditions and with different feeding approaches. In terms of physiological parameters, no obvious differences could be observed between the set of CalB producer strains based on different promoters, although the titers achieved were notably different, suggesting that CalB production does not affect the cell fitness.

Due to the natural growth-decoupled regulation of P_DH_, switching between high and low feeding rates was a successful and relatively simple strategy to boost the CalB production. As a result, a single cycle of biomass growth and expression induction through pseudo-starving allowed achieving the highest productivities for PDH-C clone. In particular, this approach obtained 3.6-fold higher Q_p_ than the GAP-C fed-batch cultivation at low µ, while it was really close to the optimal GAP-C fed-batch cultivation. On the other hand, three biomass-induction cycles with PDH-C provided the highest titer compared with all GAP-C cultivations, being in the range of 2.3- to 3.6-fold times higher.

When applying pseudo-starving conditions together with osmotic shock to PDH-C, neither in shake-flask nor in fed-batch cultivations a remarkable increase in protein production was achieved, despite P_DH_ being natively related to stress-damage repair. Therefore, it was conjectured that the majority of stress mechanisms were already activated by the protein over-expression and carbon source deprivation.

Exploiting the derepressed regulation of P_DH_, growth-decoupled fed-batch cultivation strategies were successfully implemented, achieving a higher production of CalB than GAP-C. Although PDF-C still showed better results in terms of final titer and overall productivities, the novel promoter P_DH_ presents an innovative and promising alternative to methanol-based bioprocesses, especially for toxic, low- and high-value added products. In addition, P_DH_ is operationally more advantageous than other methanol-free promoters since it can be tightly controlled only by adjusting the feeding rate. To sum up, this expression system increases further the versatility of *K. phaffii* and expands its toolbox for RPP, being a step forward to achieve the circular bioeconomy transition.

## Data Availability

The transcriptome data presented in the study are deposited in the European Nucleotide Archive (ENA) Database repository, under accession number PRJEB58889.
