# Supplementary material for: Enabling growth-decoupled Komagataella phaffii recombinant protein production based on the methanol-free PDH promoter
Source: Front Bioeng Biotechnol. 2023 Mar 23;11:1130583. doi: 10.3389/fbioe.2023.1130583 (PMC10076887; doi:10.3389/fbioe.2023.1130583)
Supplement: Supplementary file 1 [file DataSheet1.pdf]

Supplementary Material

1.1 Supplementary data

PCRs for promoter sequence identification

The different truncations of the promoter sequence were made by PCR. As template the synthetic DNA of the *HSP12* homologous promoter region was used. Primers were set approximately every 50 bp and ordered with appropriate overhangs for Gibson Cloning (length > 20bp, Tm > 60°C). A primer list and the truncated sequences can be found in the Supplementary Table S1 and S2, respectively. After verification of the PCR amplicon length by agarose gel electrophoresis, DNA fragments were purified using a commercially available silica column-based kit.

Supplementary Table S1: Primer sequences used for P_{HSP12} truncations.

Primer ID	Sequence 5' - 3'
PDH_1000_fw	CGAAGAAGAGACCCCTAGGCCGTACGACAGGATAAAGAACGACCGGAACCAATACGGG
PDH_950_fw	CGAAGAAGAGACCCCTAGGCCGTACGACAGTGTTGGTTTGGATTCTTGACGTGAAGAAG
PDH_900_fw	CGAAGAAGAGACCCCTAGGCCGTACGACAGTTGATAAGGATATGGCGTCACTGAGTTG
PDH_850_fw	CGAAGAAGAGACCCCTAGGCCGTACGACAGGTTGGGTCAGGAGTTAATTGATTCACCTC
PDH_800_fw	CGAAGAAGAGACCCCTAGGCCGTACGACAGGTTTTCAGATGGGCATCTTTCTTGACGG
PDH_750_fw	CGAAGAAGAGACCCCTAGGCCGTACGACAGTGTTTGAGCCTGTACGCGGTTACTTCTG
PDH_665_fw	CGAAGAAGAGACCCCTAGGCCGTACGACAGTGATTAGAAGGTGAATGAGGGAGTCACC
PDH_600_fw	CGAAGAAGAGACCCCTAGGCCGTACGACAGAAGTAAAGTAGCACTTCTGGCAGGTTAG
PDH_550_fw	CGAAGAAGAGACCCCTAGGCCGTACGACAGGAAGATGGCTGGTTTTTCCTTAGTCTTG
PDH_500_fw	CGAAGAAGAGACCCCTAGGCCGTACGACAGGCGAATATTCCCCGATTAGGCAAATTAG
PDH_450_fw	CGAAGAAGAGACCCCTAGGCCGTACGACAGTGGTGATTTGTGGTAACAAATATCTATTGG
PDH_400_fw	CGAAGAAGAGACCCCTAGGCCGTACGACAGTAATAGTGGTCGTGTCATGATGATGGTG
PDH_350_fw	CGAAGAAGAGACCCCTAGGCCGTACGACAGCTTCAGTAAGAGAAGGAAGCTTGGTGAC
PDH_300_fw	CGAAGAAGAGACCCCTAGGCCGTACGACAGAATTGCTAAGCAATTGTGAGGTGTGATG
PDH_250_fw	CGAAGAAGAGACCCCTAGGCCGTACGACAGTACAACGGGGTGTTATTGTCTTCACAAG
PDH_200_fw	CGAAGAAGAGACCCCTAGGCCGTACGACAGTTGAAAGAATATTTTAGCACAGGGTGCTTG
PDH_150_fw	CGAAGAAGAGACCCCTAGGCCGTACGACAGCCATGATTGCTAAATGATGACATAGCAATC
PDH_100_fw	CGAAGAAGAGACCCCTAGGCCGTACGACAGGGACACCTTTCTTAGTATAAATGGACACTC
eGFP_PDH_rev	CCAGTGAAAAGTTCTTCTCCTTTGCTAGCCATTTCTAGTTG

Supplementary Table S2: DNA sequence of P_{HSP12} truncations.

Name	Sequence 5‘ – 3‘	Length
PDH1	GATAAAGAACGACCGGAACCAATACGGGGTTGTGCAGGTGGGAATAAATATGTTGGTTTGGATTCTTGACGTGA<br>AGAAGGTATTCTAGTCGATGAAGTGGTTGATAAGGATATGGCGTCACTGAGTTGTTTTCTTTTCCTATGTTGCGGT<br>GTTGGGTCAGGAGTTAATTGATTCACCTCCATAACTCTGGAATTTCTTGAATGTGGGGTTTTCAGATGGGCATCTT<br>TCTTGACGGGGTTGTGAGTAACGGAGGAACCTGGTGTCTTGGGTGTGAACGGTGTTTGAGCCTGTACGCGGTTAC<br>TTCTGGGCGGAGTACTCGGAGTCATGAGAGCCATTGATTAGAAGGTGAATGAGGGAGTCACCACTCTAAGCAAA<br>CAAAATGAGGTCGAAGCAAAAAATAAAGTAAAGTAGCACTTCTGGCAGGTTAGATCAAAGAGTGACGGGAGATT<br>TGAAGATGGCTGGTTTTTCCTTAGTCTTGGAAGAGGTTTGTGTGGGTATCAGCGAATATTCCCCGATTAGGCAAAT<br>TAGTTGCATTGAAATTAACACGACATGGTGATTTGTGGTAACAAATATCTATTGGTGGTTGGTGTGTGGGTGTAAT<br>AGTGGTCGTGTCATGATGATGGTGTTCAGGTGTTGTCATAGATCGGTCTTCAGTAAGAGAAGGAAGCTTGGTGAC<br>GATCACAGCTATGATGTAATAGAAATTGCTAAGCAATTGTGAGGTGTGATGTATTTTGCAGAGCAATTGTGCGGT<br>ACAACGGGGTGTTATTGTCTTCACAAGGCATTTATTGCGAATTTCGTAGTTGAAAGAATATTTAGCACAGGGTGC<br>TTGACCCCTATTGTTGCTCGCTAAACCATGATTGCTAAATGATGACATAGCAATCACTTTACTAAGATTGCTATAA<br>GGACACCTTTCTTAGTATAAATGGACACTCTTTTCCCCTGCTAAACTTCTTTTATTTTTCACACTTAAACAGTTACA<br>AAACACAAACACAACTAGAA	1000
PDH3	TTGATAAGGATATGGCGTCACTGAGTTGTTTTCTTTTCCTATGTTGCGGTGTTGGGTCAGGAGTTAATTGATTCAC<br>CTCCATAACTCTGGAATTTCTTGAATGTGGGGTTTTCAGATGGGCATCTTTCTTGACGGGGTTGTGAGTAACGGAG<br>GAACCTGGTGTCTTGGGTGTGAACGGTGTTTGAGCCTGTACGCGGTTACTTCTGGGCGGAGTACTCGGAGTCATG<br>AGAGCCATTGATTAGAAGGTGAATGAGGGAGTCACCACTCTAAGCAAACAAAATGAGGTCGAAGCAAAAAATAA<br>AGTAAAGTAGCACTTCTGGCAGGTTAGATCAAAGAGTGACGGGAGATTTGAAGATGGCTGGTTTTTCCTTAGTCT<br>TGGAAGAGGTTTGTGTGGGTATCAGCGAATATTCCCCGATTAGGCAAATTAGTTGCATTGAAATTAACACGACAT<br>GGTGATTTGTGGTAACAAATATCTATTGGTGGTTGGTGTGTGGGTGTAATAGTGGTCGTGTCATGATGATGGTGTT<br>CAGGTGTTGTCATAGATCGGTCTTCAGTAAGAGAAGGAAGCTTGGTGACGATCACAGCTATGATGTAATAGAAAT<br>TGCTAAGCAATTGTGAGGTGTGATGTATTTTGCAGAGCAATTGTGCGGTACAACGGGGTGTTATTGTCTTCACAA<br>GGCATTTATTGCGAATTTCGTAGTTGAAAGAATATTTAGCACAGGGTGCTTGACCCCTATTGTTGCTCGCTAAAC<br>CATGATTGCTAAATGATGACATAGCAATCACTTTACTAAGATTGCTATAAGGACACCTTTCTTAGTATAAATGGAC<br>ACTCTTTTCCCCTGCTAAACTTCTTTTATTTTTCACACTTAAACAGTTACAAAACACAAACACAACTAGAA	900
PDH4	GTTGGGTCAGGAGTTAATTGATTCACCTCCATAACTCTGGAATTTCTTGAATGTGGGGTTTTCAGATGGGCATCTT<br>TCTTGACGGGGTTGTGAGTAACGGAGGAACCTGGTGTCTTGGGTGTGAACGGTGTTTGAGCCTGTACGCGGTTAC<br>TTCTGGGCGGAGTACTCGGAGTCATGAGAGCCATTGATTAGAAGGTGAATGAGGGAGTCACCACTCTAAGCAAA<br>CAAAATGAGGTCGAAGCAAAAAATAAAGTAAAGTAGCACTTCTGGCAGGTTAGATCAAAGAGTGACGGGAGATT<br>TGAAGATGGCTGGTTTTTCCTTAGTCTTGGAAGAGGTTTGTGTGGGTATCAGCGAATATTCCCCGATTAGGCAAAT<br>TAGTTGCATTGAAATTAACACGACATGGTGATTTGTGGTAACAAATATCTATTGGTGGTTGGTGTGTGGGTGTAAT<br>AGTGGTCGTGTCATGATGATGGTGTTCAGGTGTTGTCATAGATCGGTCTTCAGTAAGAGAAGGAAGCTTGGTGAC<br>GATCACAGCTATGATGTAATAGAAATTGCTAAGCAATTGTGAGGTGTGATGTATTTTGCAGAGCAATTGTGCGGT<br>ACAACGGGGTGTTATTGTCTTCACAAGGCATTTATTGCGAATTTCGTAGTTGAAAGAATATTTAGCACAGGGTGC<br>TTGACCCCTATTGTTGCTCGCTAAACCATGATTGCTAAATGATGACATAGCAATCACTTTACTAAGATTGCTATAA<br>GGACACCTTTCTTAGTATAAATGGACACTCTTTTCCCCTGCTAAACTTCTTTTATTTTTCACACTTAAACAGTTACA<br>AAACACAAACACAACTAGAA	850
PDH5	GTTTTCAGATGGGCATCTTTCTTGACGGGGTTGTGAGTAACGGAGGAACCTGGTGTCTTGGGTGTGAACGGTGTTT<br>GAGCCTGTACGCGGTTACTTCTGGGCGGAGTACTCGGAGTCATGAGAGCCATTGATTAGAAGGTGAATGAGGGA<br>GTCACCACTCTAAGCAAACAAAATGAGGTCGAAGCAAAAAATAAAGTAAAGTAGCACTTCTGGCAGGTTAGATC<br>AAAGAGTGACGGGAGATTTGAAGATGGCTGGTTTTTCCTTAGTCTTGGAAGAGGTTTGTGTGGGTATCAGCGAAT<br>ATTCCCCGATTAGGCAAATTAGTTGCATTGAAATTAACACGACATGGTGATTTGTGGTAACAAATATCTATTGGTG<br>GTTGGTGTGTGGGTGTAATAGTGGTCGTGTCATGATGATGGTGTTCAGGTGTTGTCATAGATCGGTCTTCAGTAAG<br>AGAAGGAAGCTTGGTGACGATCACAGCTATGATGTAATAGAAATTGCTAAGCAATTGTGAGGTGTGATGTATTTT<br>GCAGAGCAATTGTGCGGTACAACGGGGTGTTATTGTCTTCACAAGGCATTTATTGCGAATTTCGTAGTTGAAAGA<br>ATATTTAGCACAGGGTGCTTGACCCCTATTGTTGCTCGCTAAACCATGATTGCTAAATGATGACATAGCAATCAC<br>TTTACTAAGATTGCTATAAGGACACCTTTCTTAGTATAAATGGACACTCTTTTCCCCTGCTAAACTTCTTTTATTTT<br>TCACACTTAAACAGTTACAAAACACAAACACAACTAGAA	793
PDH6	TGTTTGAGCCTGTACGCGGTTACTTCTGGGCGGAGTACTCGGAGTCATGAGAGCCATTGATTAGAAGGTGAATGA<br>GGGAGTCACCACTCTAAGCAAACAAAATGAGGTCGAAGCAAAAAATAAAGTAAAGTAGCACTTCTGGCAGGTTA<br>GATCAAAGAGTGACGGGAGATTTGAAGATGGCTGGTTTTTCCTTAGTCTTGGAAGAGGTTTGTGTGGGTATCAGC<br>GAATATTCCCCGATTAGGCAAATTAGTTGCATTGAAATTAACACGACATGGTGATTTGTGGTAACAAATATCTATT<br>GGTGGTTGGTGTGTGGGTGTAATAGTGGTCGTGTCATGATGATGGTGTTCAGGTGTTGTCATAGATCGGTCTTCAG<br>TAAGAGAAGGAAGCTTGGTGACGATCACAGCTATGATGTAATAGAAATTGCTAAGCAATTGTGAGGTGTGATGT<br>ATTTTGCAGAGCAATTGTGCGGTACAACGGGGTGTTATTGTCTTCACAAGGCATTTATTGCGAATTTCGTAGTTGA<br>AAGAATATTTAGCACAGGGTGCTTGACCCCTATTGTTGCTCGCTAAACCATGATTGCTAAATGATGACATAGCA<br>ATCACTTTACTAAGATTGCTATAAGGACACCTTTCTTAGTATAAATGGACACTCTTTTCCCCTGCTAAACTTCTTTT<br>ATTTTTCACACTTAAACAGTTACAAAACACAAACACAACTAGAA	722

PDH7	TGATTAGAAGGTGAATGAGGGAGTCACCACTCTAAGCAAACAAAATGAGGTCGAAGCAAAAAATAAAGTAAAGT<br>AGCACTTCTGGCAGGTTAGATCAAAGAGTGACGGGAGATTTGAAGATGGCTGGTTTTTCCTTAGTCTTGGAAGAG<br>GTTTGTGTGGGTATCAGCGAATATTCCCCGATTAGGCAAATTAGTTGCATTGAAATTAACACGACATGGTGATTTG<br>TGGTAACAAATATCTATTGGTGGTTGGTGTGTGGGTGTAATAGTGGTCGTGTCATGATGATGGTGTTCAGGTGTTG<br>TCATAGATCGGTCTTCAGTAAGAGAAGGAAGCTTGGTGACGATCACAGCTATGATGTAATAGAAATTGCTAAGCA<br>ATTGTGAGGTGTGATGTATTTTGCAGAGCAATTGTGCGGTACAACGGGGTGTTATTGTCTTCACAAGGCATTTATT<br>GCGAATTTCGTAGTTGAAAGAATATTTTAGCACAGGGTGCTTGACCCCTATTGTTGCTCGCTAAACCATGATTGCT<br>AAATGATGACATAGCAATCACTTTACTAAGATTGCTATAAGGACACCTTTCTTAGTATAAATGGACACTCTTTTCC<br>CCTGCTAAACTTCTTTTATTTTTCACACTTAAACAGTTACAAAACACAAACACAACTAGAA	665
PDH8	AAGTAAAGTAGCACTTCTGGCAGGTTAGATCAAAGAGTGACGGGAGATTTGAAGATGGCTGGTTTTTCCTTAGTC<br>TTGGAAGAGGTTTGTGTGGGTATCAGCGAATATTCCCCGATTAGGCAAATTAGTTGCATTGAAATTAACACGACA<br>TGGTGATTTGTGGTAACAAATATCTATTGGTGGTTGGTGTGTGGGTGTAATAGTGGTCGTGTCATGATGATGGTGT<br>TCAGGTGTTGTCATAGATCGGTCTTCAGTAAGAGAAGGAAGCTTGGTGACGATCACAGCTATGATGTAATAGAAA<br>TTGCTAAGCAATTGTGAGGTGTGATGTATTTTGCAGAGCAATTGTGCGGTACAACGGGGTGTTATTGTCTTCACAA<br>GGCATTTATTGCGAATTTCGTAGTTGAAAGAATATTTTAGCACAGGGTGCTTGACCCCTATTGTTGCTCGCTAAAC<br>CATGATTGCTAAATGATGACATAGCAATCACTTTACTAAGATTGCTATAAGGACACCTTTCTTAGTATAAATGGAC<br>ACTCTTTTCCCCTGCTAAACTTCTTTTATTTTTCACACTTAAACAGTTACAAAACACAAACACAACTAGAA	600
PDH11	TGGTGATTTGTGGTAACAAATATCTATTGGTGGTTGGTGTGTGGGTGTAATAGTGGTCGTGTCATGATGATGGTGT<br>TCAGGTGTTGTCATAGATCGGTCTTCAGTAAGAGAAGGAAGCTTGGTGACGATCACAGCTATGATGTAATAGAAA<br>TTGCTAAGCAATTGTGAGGTGTGATGTATTTTGCAGAGCAATTGTGCGGTACAACGGGGTGTTATTGTCTTCACAA<br>GGCATTTATTGCGAATTTCGTAGTTGAAAGAATATTTTAGCACAGGGTGCTTGACCCCTATTGTTGCTCGCTAAAC<br>CATGATTGCTAAATGATGACATAGCAATCACTTTACTAAGATTGCTATAAGGACACCTTTCTTAGTATAAATGGAC<br>ACTCTTTTCCCCTGCTAAACTTCTTTTATTTTTCACACTTAAACAGTTACAAAACACAAACACAACTAGAA	450
PDH12	TAATAGTGGTCGTGTCATGATGATGGTGTTCAGGTGTTGTCATAGATCGGTCTTCAGTAAGAGAAGGAAGCTTGG<br>TGACGATCACAGCTATGATGTAATAGAAATTGCTAAGCAATTGTGAGGTGTGATGTATTTTGCAGAGCAATTGTG<br>CGGTACAACGGGGTGTTATTGTCTTCACAAGGCATTTATTGCGAATTTCGTAGTTGAAAGAATATTTTAGCACAGG<br>GTGCTTGACCCCTATTGTTGCTCGCTAAACCATGATTGCTAAATGATGACATAGCAATCACTTTACTAAGATTGCT<br>ATAAGGACACCTTTCTTAGTATAAATGGACACTCTTTTCCCCTGCTAAACTTCTTTTATTTTTCACACTTAAACAGT<br>TACAAAACACAAACACAACTAGAA	403
PDH13	CTTCAGTAAGAGAAGGAAGCTTGGTGACGATCACAGCTATGATGTAATAGAAATTGCTAAGCAATTGTGAGGTGT<br>GATGTATTTTGCAGAGCAATTGTGCGGTACAACGGGGTGTTATTGTCTTCACAAGGCATTTATTGCGAATTTCGTA<br>GTTGAAAGAATATTTTAGCACAGGGTGCTTGACCCCTATTGTTGCTCGCTAAACCATGATTGCTAAATGATGACAT<br>AGCAATCACTTTACTAAGATTGCTATAAGGACACCTTTCTTAGTATAAATGGACACTCTTTTCCCCTGCTAAACTT<br>CTTTTATTTTTCACACTTAAACAGTTACAAAACACAAACACAACTAGAA	352
PDH14	AATTGCTAAGCAATTGTGAGGTGTGATGTATTTTGCAGAGCAATTGTGCGGTACAACGGGGTGTTATTGTCTTCAC<br>AAGGCATTTATTGCGAATTTCGTAGTTGAAAGAATATTTTAGCACAGGGTGCTTGACCCCTATTGTTGCTCGCTAA<br>ACCATGATTGCTAAATGATGACATAGCAATCACTTTACTAAGATTGCTATAAGGACACCTTTCTTAGTATAAATGG<br>ACACTCTTTTCCCCTGCTAAACTTCTTTTATTTTTCACACTTAAACAGTTACAAAACACAAACACAACTAGAA	301
PDH15	TACAACGGGGTGTTATTGTCTTCACAAGGCATTTATTGCGAATTTCGTAGTTGAAAGAATATTTTAGCACAGGGTG<br>CTTGACCCCTATTGTTGCTCGCTAAACCATGATTGCTAAATGATGACATAGCAATCACTTTACTAAGATTGCTATA<br>AGGACACCTTTCTTAGTATAAATGGACACTCTTTTCCCCTGCTAAACTTCTTTTATTTTTCACACTTAAACAGTTAC<br>AAAACACAAACACAACTAGAA	250
PDH16	TTGAAAGAATATTTTAGCACAGGGTGCTTGACCCCTATTGTTGCTCGCTAAACCATGATTGCTAAATGATGACATA<br>GCAATCACTTTACTAAGATTGCTATAAGGACACCTTTCTTAGTATAAATGGACACTCTTTTCCCCTGCTAAACTTC<br>TTTTATTTTTCACACTTAAACAGTTACAAAACACAAACACAACTAGAA	200
PDH18	GGACACCTTTCTTAGTATAAATGGACACTCTTTTCCCCTGCTAAACTTCTTTTATTTTTCACACTTAAACAGTTACA<br>AAACACAAACACAACTAGAA	97

Cloning of the truncated promoter sequences

The different promoter truncations (purified PCR amplicons) were then cloned into an entry plasmid by isothermal assembly following Gibson (figure 1) (Gibson, 2009; Gibson et al., 2009). The entry plasmid contained a 1100 bp homologous region to the 3'UTR of the *K. phaffii ARG4* gene to ensure comparability of the different constructs by targeted integration, eGFP as a reporter gene and the *AOX1* transcription terminator for eGFP transcription termination. Additionally, a zeocin™ resistance cassette was present to enable selection and a pUC origin of replication for plasmid maintenance and propagation in *E. coli*.

For the assembly, 100 ng of the linear entry plasmid (linearized between 3'UTR of *ARG4* gene and eGFP coding sequence) were mixed with 50-80 ng of the PCR amplicon (promoter truncation) in a total volume of 5 µL. The DNA was mixed with 15 µL of self-made master mix and incubated at 50°C for one hour. After assembly, 2.5 µL of the reaction mixture was introduced into *E. coli* for propagation of newly assembled plasmids, transformants were selected on LB plates supplemented with an appropriate amount of Zeocin. Correct assembly of the plasmids was verified by Sanger sequencing (service provided by Microsynth AG).

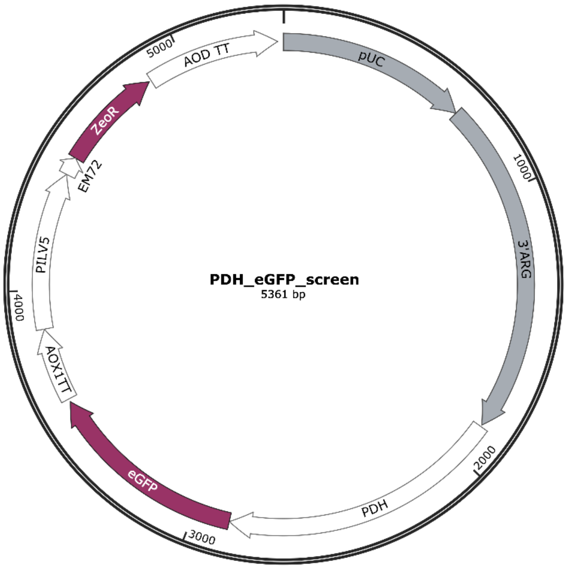

Supplementary Figure S1: Plasmid Map of the screening plasmid

K. phaffii transformation with the truncated promoter sequences

Plasmids were then linearized by *Smi*I and cells of the *K. phaffii* strain BSY11dKU70 were transformed with 1 µg linearized DNA. Preparation and transformation of electrocompetent *K. phaffii* cells was performed according to Lin-Cereghino et al. (2005). Selection was done on zeocin containing YPD agar plates.

Cultivation and eGFP measurement of the truncated promoter clones

Seven clones per each construction were then cultivated in 96-well plates for 6 days. Starting volume was 250 µL buffered minimal media with either 1% (w/v) glucose (BMD1) or 1% (w/v) glycerol (BMG1). After each 24 h 40 µL of culture were discarded, 10 µL were used for eGFP and cell density measurement in a plate reader (eGFP: 488 nm absorption, 507 nm emission; cell density: 600 nm), and 100 µL of fresh BMD1 or BMG1 were added to the culture to be as near as possible to a continuous cultivation as it is possible employing deep-well plates.

Supplementary Table S2: List of primers used for qPCR to determine gene dosage of PDH-C by amplifying the *Zeocine* gene (Zeo^R) in the expression cassette and Arginine gene (*ARG4*) as a housekeeping (Krainer et al., 2012).

Primer ID	Sequence 5' - 3'
Zeo^R_fw	GACTCGGTTTCTCCCGTGACT
Zeo^R_rev	CTGCGGAGATGAACAGGGTAA
*ARG4*_fw	TCCTCCGGTGGCAGTTCTT
*ARG4*_rev	TCCATTGACTCCCGTTTTGAG

Supplementary Table S3: Results of the copy number determination of three P_{DH}-based candidate clones by pPCR. The selected clone is labelled in grey, re-named as PDH-C.

Strain	Copy Number	Average	SD
P_{DH}-CalB A5	1	1.10	0.02
P_{DH}-CalB C2	2	1.9	0.05
P_{DH}-CalB D2	1	1.2	0.02
Reference strain	1	1.00	0.00

1.2 Supplementary Figures

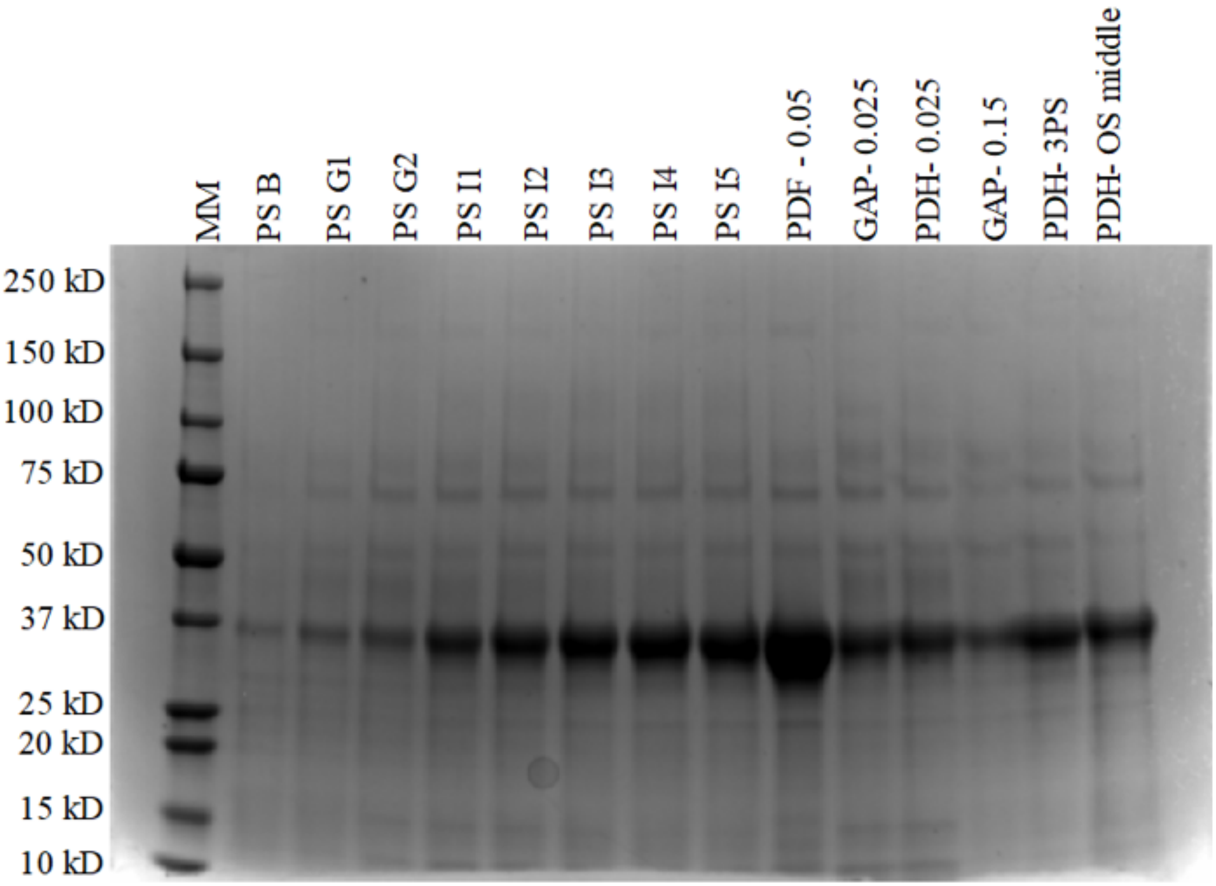

Supplementary Figure S2. SDS-PAGE comparison of all fed-batches performed with PDH-C, GAP-C and PDF-C. MM corresponds to the molecular marker Precision Plus Protein™ All Blue Prestained Protein Standards. B means the sample taken at the end of the batch phase, G at the end of the exponential growth-phase and I after applying the pseudo-starving conditions, while the numbers represent samples.

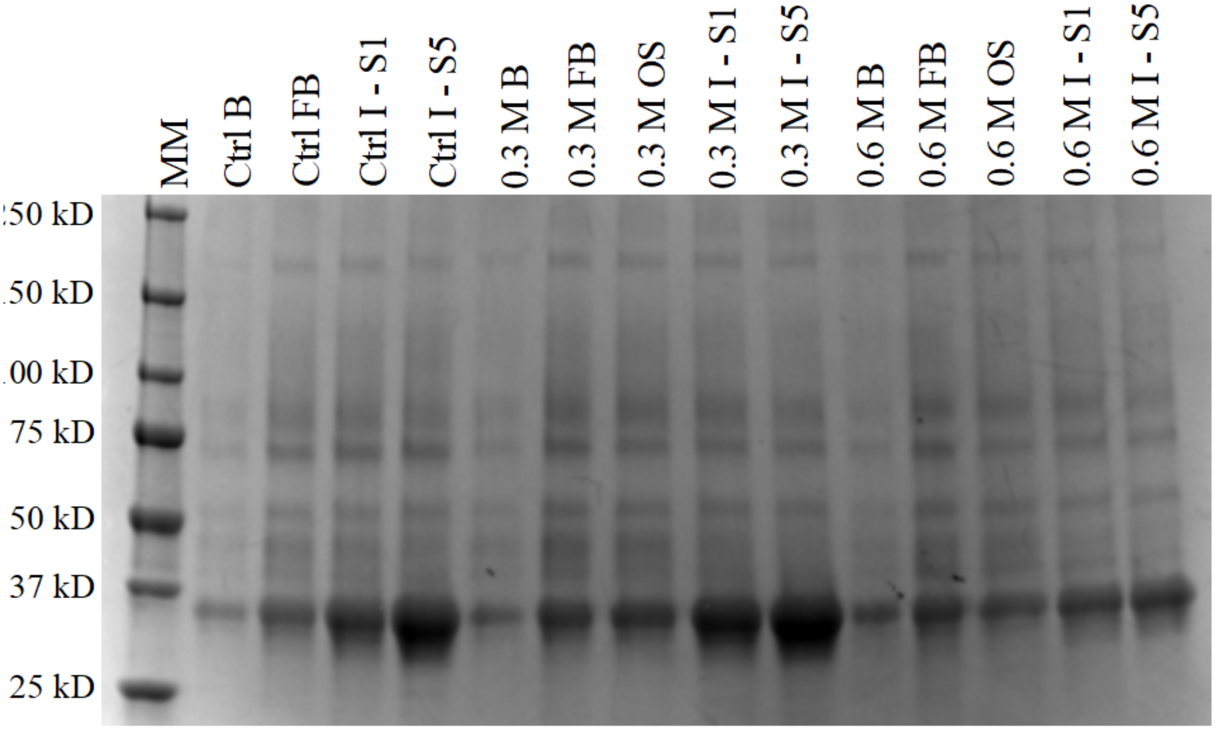

Supplementary Figure S3. SDS-PAGE comparison of osmotic shock fed-batch with simple pseudo-starving fed-batches. MM corresponds to the molecular marker Precision Plus Protein™ All Blue Prestained Protein Standards. B means the sample taken at the end of the batch phase, FB at the end of the exponential growth-phase, OS after applying the osmotic shock and S1 or S5 represent de the samples 1 and 5 from the induction phase.

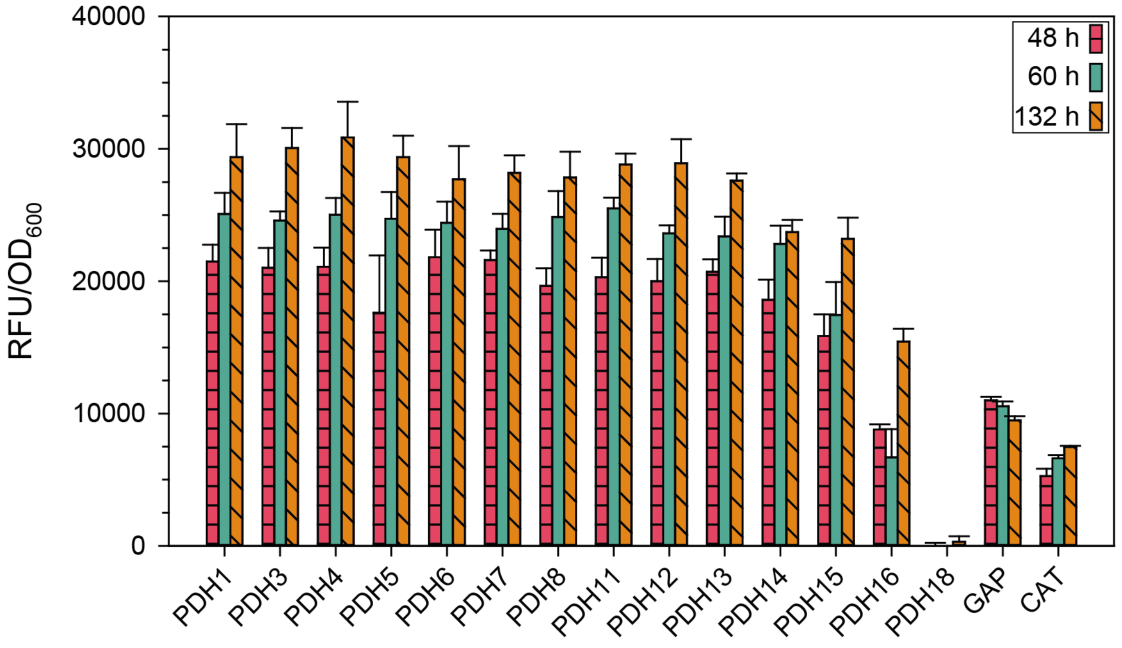

Supplementary Figure S4. Expression levels of the different PDH promoter truncations in raw fluorescence units normalized by cell density. Cells were cultivated in biological 7-fold replicates which represent the standard deviation, taking samples after 58, 60 and 132 h of cultivating with glucose as a sole carbon source. As controls, the same construct was used with P_{GAP} and P_{DC} instead of P_{DH}.-

1.3 References

Gibson, D. G. (2009). Synthesis of DNA fragments in yeast by one-step assembly of overlapping oligonucleotides. *Nucleic Acids Res.* 37, 6984–6990. doi: 10.1093/NAR/GKP687.

Gibson, D. G., Young, L., Chuang, R. Y., Venter, J. C., Hutchison, C. A., and Smith, H. O. (2009). Enzymatic assembly of DNA molecules up to several hundred kilobases. *Nat. Methods* 6, 343–345. doi: 10.1038/nmeth.1318.

Krainer, F. W., Dietzsch, C., Hajek, T., Herwig, C., Spadiut, O., and Glieder, A. (2012). Recombinant protein expression in *Pichia pastoris* strains with an engineered methanol utilization pathway. *Microb. Cell Fact.* 11, 22. doi: 10.1186/1475-2859-11-22.

Lin-Cereghino, J., Wong, W. W., Xiong, S., Giang, W., Luong, L. T., Vu, J., et al. (2005). Condensed protocol for competent cell preparation and transformation of the methylotrophic yeast *Pichia pastoris*. *Biotechniques* 38, 44–48. doi: 10.2144/05381BM04.
